# Electrolyte Structure Governs Formate Oxidation in Water-in-Salt Systems

**DOI:** 10.1021/jacs.5c19298

**Published:** 2026-03-19

**Authors:** Katharina Trapp, Soracha Kosasang, Johannes Ingenmey, Dario Gomez Vazquez, Manuel Reiter, Mathieu Salanne, Maria R. Lukatskaya

**Affiliations:** † Electrochemical Energy Systems Laboratory, Department of Mechanical and Process Engineering, 111842 ETH Zurich, Zurich 8092, Switzerland; ‡ 27063 Réseau sur le Stockage Electrochimique de l’Energie (RS2E), CNRS FR3459, Amiens 80039, France; § Physicochimie des Électrolytes et Nanosystemes Interfaciaux, Sorbonne Université, CNRS, PHENIX, Paris F-75005, France; ∥ Institut Universitaire de France (IUF), Paris 75231, France

## Abstract

We disentangle reactant concentration from local structural effects in water-in-salt electrolytes using the formate oxidation reaction on Pt. First, we observe that formate oxidation currents plateau at high concentrations. Using molecular dynamics and NMR spectroscopy, we attribute this observation to ion clustering of the kosmotropic formate reactant, which reduces conductivity and impedes reactant transport. Then, we demonstrate that this limitation can be overcome by introducing a chaotropic anion (perchlorate) that disrupts clustering and facilitates a further increase in formate oxidation currents. However, when perchlorate is introduced in excess, the hydrogen-bonding network is disrupted, which leads to hindered proton transport, local acidification, and enhanced CO poisoning, as evidenced by SEIRAS. Our findings demonstrate a direct link between bulk electrolyte structure and catalytic activity, which can be used to enhance catalytic performance at high reactant concentrations.

## Graphical abstract

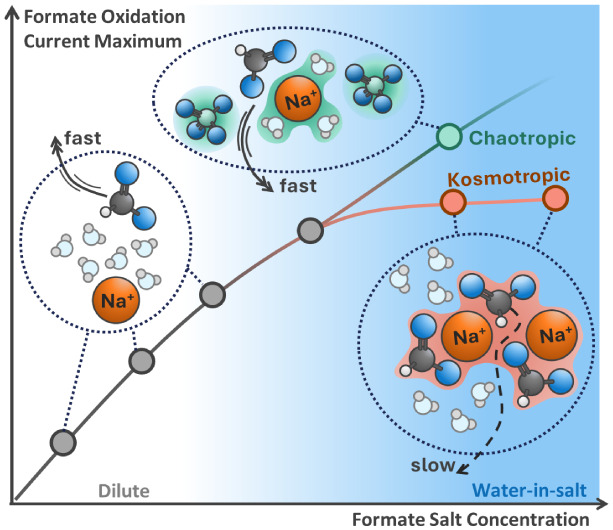

## Introduction

In electrocatalytic reactions, the electrolyte plays a crucial role in defining key properties such as interfacial pH and buffering capacity, double layer structure, and the diffusion of reactants and products.^1,2^ Additionally, electrolyte-adsorbate interactions,^3,4^ and surface-site blocking^2,5^ directly influence catalytic performance, making the choice of electrolyte critical. Changing the electrolyte concentration represents one of the tuning knobs for electrolyte engineering. While electrolytes with low or intermediate concentrations (usually ≤ 1 mol/L) are standard practice, the use of high salt concentrations remains underexplored in electrocatalysis. This is due to several challenges: established electrochemical models and mathematical approximations are no longer valid at high salt concentrations and, additionally, the solubility of many reactants is reduced in these conditions (*e.g.,* CO_2_).^6–8^

Recent studies highlight the potential of concentrated electrolytes.^6,9–11^ A reduced water-to-ion ratio alters the electrolyte structure, which in turn influences the electrocatalytic performance.^6,9–11^ The highest concentrations are reached in water-in-salt (WIS) electrolytes, where the cation’s primary solvation shell remains incomplete due to limited water amounts.^8^ Initially introduced for battery applications, they hinder the hydrogen evolution reaction and increase the electrochemical stability window.^12–15^ More recently, WIS electrolytes were employed in the electrochemical CO_2_ reduction and ammonia synthesis, enabling improved faradaic efficiencies toward target products.^6,9–11^ However, the impact of high salt concentrations on electrocatalytic processes remains poorly understood. Therefore, systematic studies of concentration effects in electrocatalytic model reactions are needed for advancing the knowledge-based electrolyte design.

As a model reaction for the electrooxidation of small organic molecules, the formic acid/formate oxidation (FOR) is applied in direct formic acid/formate fuel cells. Mechanistic studies of FOR, predominantly conducted in dilute electrolytes, suggest that both the direct and indirect reaction pathway proceed *via* an adsorbed formate intermediate, even when formic acid is used as the reactant.^16–25^ While the indirect pathway leads to CO poisoning, the direct pathway proceeds rapidly, with the oxidation of the adsorbed formate intermediate as rate-limiting step.^19,22^ While these studies provide insight into the reaction pathway, they overwhelmingly focus on dilute electrolytes^16–25^ and studies at higher concentrations remain scarce. Most work involving high-concentration electrolytes target strongly acidic environments, where FOR performance is substantially diminished, masking effects of the altered electrolyte structure. Under acidic conditions, FOR current densities stagnate at formic acid concentrations >1–2 M and become negligible in 26 M formic acid due to severe CO poisoning and the unfavorable formic acid–formate equilibrium.^26–29^ In contrast, the alkaline nature of concentrated formate electrolytes mitigates equilibrium limitations and their feasibility for FOR has been demonstrated in fuel cells (3–7 M formate).^30–32^ In such electrolytes, changes in FOR performance and their relationship to solvation structure, water activity and the nature of supporting electrolyte salts remains unexplored, despite their potentially crucial impact. Establishing structure–performance relationships in these high-concentration systems is therefore essential for advancing electrolyte engineering. Given its well-understood behavior in dilute solutions, FOR provides an ideal platform for this work, enabling a focused investigation into the distinct effects of highly concentrated electrolytes on electrocatalysis.

Herein, we decouple the influence of high reactant concentration from the structural effects of water-in-salt (WIS) electrolytes by investigating the formate oxidation reaction (FOR) on polycrystalline Pt in two distinct electrolyte series. First, to probe the impact of concentration, we examined Na­(HCOO)·y H_2_O electrolytes, varying the water content (y) from 555 (dilute) down to 4 (WIS regime). Second, to isolate structural effects, we studied a complementary WIS series, Na­[(HCOO)_x_(ClO_4_)_1–x_]·4 H_2_O (x = 0.007–0.75), which introduces a chaotropic anion ClO_4_^–^ while maintaining a constant water-to-cation ratio of 4. Importantly, the compositions were designed to match the formate molality of the first series (from 0.1 to 14 m, Table 1), allowing us to directly compare FOR activity at identical formate concentrations and thus decoupling effects of the local electrolyte structure under WIS conditions. In this framework, the effect of chaotropic perchlorate on the local electrolyte structure and its resulting influence on FOR activity is systematically examined.

**Table 1:** Molality, Molecular Denotation and Conductivity (κ) Values of the Studied Electrolytes

Formate series			Water-in-salt series		
Molecular notation	Molality	κ (mS/cm)	Molecular notation	Molality	κ (mS/cm)
Na(HCOO)·555 H_2_O	0.1 m NaHCOO	8.8	Na[(HCOO)_0.007_(ClO_4_)_0.993_]·4 H_2_O	0.1 m NaHCOO + 13.9 m NaClO_4_	112.3
Na(HCOO)·55 H_2_O	1 m NaHCOO	58.6	Na[(HCOO)_0.07_(ClO_4_)_0.93_]·4 H_2_O	1 m NaHCOO + 13 m NaClO_4_	143.3
Na(HCOO)·16 H_2_O	3.5 m NaHCOO	122.0	Na[(HCOO)_0.25_(ClO_4_)_0.75_]·4 H_2_O	3.5 m NaHCOO + 10.5 m NaClO_4_	130.5
Na(HCOO)·8 H_2_O	7 m NaHCOO	123.7	Na[(HCOO)_0.5_(ClO_4_)_0.5_]·4 H_2_O	7 m NaHCOO + 7 m NaClO_4_	107.3
Na(HCOO)·5 H_2_O	10.5 m NaHCOO	99.7	Na[(HCOO)_0.75_(ClO_4_)_0.25_]·4 H_2_O	10.5 m NaHCOO + 3.5 m NaClO_4_	90.7
Na(HCOO)·4 H_2_O	14 m NaHCOO	77.8	Na(HCOO)·4 H_2_O	14 m NaHCOO	77.8

## Results and Discussion

### Electrolyte Structure

An increase in electrolyte concentration leads to modified local chemical environments.^33^ To probe changes in the sodium formate electrolyte structure upon an increase in concentration and addition of NaClO_4_, we used a combination of Fourier transform infrared (FTIR) spectroscopy, nuclear magnetic resonance spectroscopy (NMR), and molecular dynamics (MD) simulations (Figure 1).

**Figure 1:**
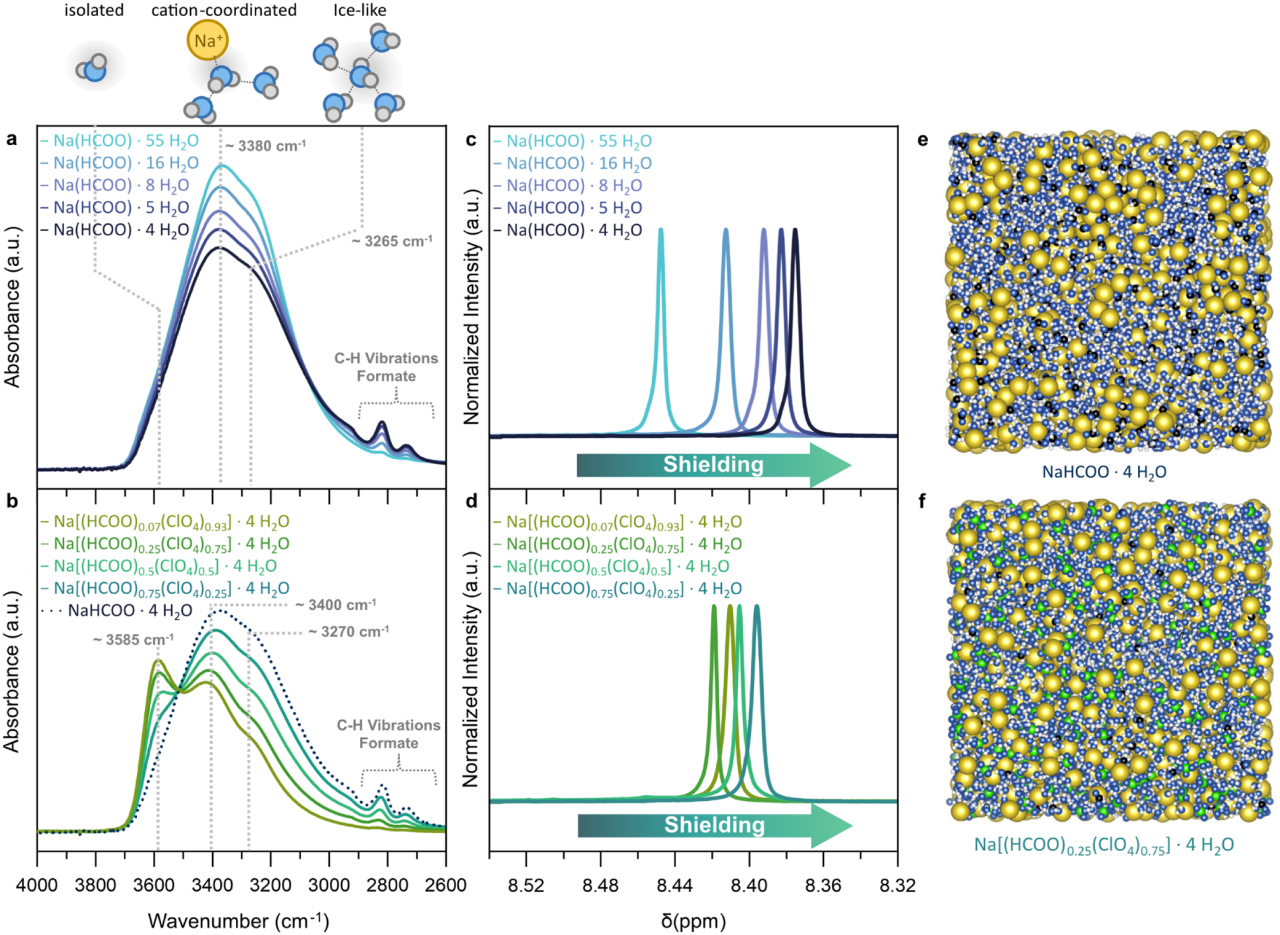
FTIR spectra of the ν­(OH) region for (a) the sodium formate electrolyte series and (b) the sodium perchlorate electrolyte series.(c–d) ^1^H NMR spectra of the sodium formate hydrogen atom for (c) the sodium formate electrolyte series and (d) the sodium perchlorate electrolyte series. (e–f) MD-simulation box of the (e) WIS electrolyte Na­(HCOO)·4H_2_O and (f) Na­[(HCOO)_0.25_(ClO_4_)_0.75_]·4 H_2_O. The yellow, blue, white, black and green spheres correspond to sodium ions, oxygen atoms, hydrogen atoms, carbon atoms and chloride atoms, respectively.

First, we investigated the water structure of the electrolytes *via* FTIR spectroscopy by evaluating the (OH) vibrations (∼3000–3600 cm^–1^, Figure 1a and b). The ν­(OH) vibration band can be deconvoluted into ν­(OH) vibrations of water molecules with different chemical environments.^34–36^ In the case of formate electrolytes Na­(HCOO)·y H_2_O with y = 55, 16, 8, 5, 4, there are no significant changes in ν­(OH) vibrations with rising formate concentration, indicating only minor modifications of the water structure. Water molecules keep 2–4 H-bonds with a dominating cation- and ice-like coordination at 3380 cm^–1^ and 3265 cm^–1^ (Figure 1a).^37^ As described by Reber et al., the observed electrolyte structure is typical for salts with “kosmotropic” anions in the Hofmeister scale.^33^ Kosmotropic anions have higher charge density and are therefore more likely to be part of the first coordination shell of cations, leaving the H-bond water structure unaffected.^33^ When replacing kosmotropic formate ions with chaotropic perchlorate to create the WIS electrolyte series Na[(HCOO)_x_(ClO_4_)_1–x_]·4 H_2_O with x = 0.07, 0.25, 0.5, 0.75, pronounced changes in the ν­(OH) vibration region are observed. The increase in isolated water molecules (∼3585 cm^–1^) and decrease of cation-coordinated (∼3400 cm^–1^) and ice-like water (∼3270 cm^–1^) with rising perchlorate content signifies the H-bond disrupting character of perchlorate (Figure 1b). The chaotropic perchlorate anion exhibits a lower charge density and is thus unfavored for the coordination with cations. Therefore, water is preferred in the cation coordination shell, leading to its isolation from other water molecules.^33,38^

^1^H NMR spectra of the formate anion show a negative chemical shift with rising formate concentration (Figure 1b), indicative of shielding (environment with higher electron density). This effect can be attributed to decreased H-bonds between the formyl hydrogen atom and water molecules as well as a closer distance to other anions, indicative of ion cluster formation.^39^ Furthermore, ^13^C NMR reveals a strong deshielding effect of the carbonyl carbon in Na­(HCOO)·4 H_2_O and Na­(HCOO)·5 H_2_O, pointing toward ion pairing, as the coordination of Na^+^ with the carbonyl oxygen of formate reduces the electron density at the carbonyl carbon atom (Figure S1). MD simulations further support the formation of ion pairs and ion clusters, as the coordination number (CN) of Na^+^ with HCOO^–^ increases in the first and second coordination shell (Table S1, Figure S2). Already in Na(HCOO)·8 H_2_O (“salt-solvate” concentration regime), each Na^+^ is coordinated by more than one formate anion while in Na­(HCOO)·5 H_2_O more than two formate anions (CN_Na–HCOO_ = 2.4) become part of the Na^+^ first solvation shell (Table S1, Figure S2b) and nearly six formate ions are found in the second shell pointing to the formation of ion clusters at concentrations ≥Na­(HCOO)·5 H_2_O.

The ^1^H NMR spectra of the WIS Na­[(HCOO)_x_(ClO_4_)_1–x_]·4 H_2_O with x = 0.07, 0.25, 0.5, 0.75 solution series show peak positions of the formyl H-atom between 8.42–8.39 ppm, which indicates reduced shielding compared to the pure formate electrolyte (Na­(HCOO)·4 H_2_O) in the WIS regime. The presence of perchlorate decreases the kosmotropic character of the electrolyte and therefore the separation of water clusters and sodium formate ions, keeping the solvation structure of formate similar to the salt-solvate regime of pure formate electrolytes. MD calculations suggest that nearly all sodium ions are coordinated with formate in their first coordination shell, as demonstrated by the CN close to the Na^+^/HCOO^–^ ratio in all perchlorate-containing electrolytes, but decreases in the second coordination shell when comparing the perchlorate-containing electrolytes to the pure formate electrolytes of the same molality (Table S1). Consequently, sodium–formate ion pairs are present in Na­[(HCOO)_x_(ClO_4_)_1–x_]·4 H_2_O with x = 0.25–0.75, but the ion cluster formation is reduced. The electrolyte Na­[(HCOO)_0.75_(ClO_4_)_0.25_]·4 H_2_O has one less formate ion in its second coordination shell of Na^+^ (5.6 vs 4.6, Table S1, Figure S3), and a slight reduction of CN_Na–HCOO_ in the first coordination shell when comparing it to the pure Na­(HCOO)·5 H_2_O electrolyte with equal formate molality (10.5 m NaHCOO, Table 1). Besides the interactions of sodium and formate, sodium–perchlorate ion pairs are also suggested by MD simulations (Figure S4). This is consistent with FTIR spectroscopy, which shows a redshift of the Cl–O asymmetric stretching vibration (1100 cm^–1^ to 1086 cm^–1^) with increasing perchlorate concentration, previously assigned to the formation of solvent separated Na^+^–ClO_4_^–^ ion pairs (Figure S5).^40^

The MD simulation boxes show the resulting overall structure of the WIS electrolytes with high formate (Figure 1e) or high perchlorate concentration (Figure 1f): The formate WIS electrolyte is dominated by strong sodium-formate cluster formation creating water domains with a H-bond network similar to dilute conditions and a strongly altered solvation environment of formate ions. The addition of perchlorate leads to a homogeneous distribution of sodium ions throughout the electrolyte and a disrupted hydrogen bond network. A high perchlorate/formate ratio retains sodium-formate ion pairs but reduces Na^+^–HCOO^–^ cluster formation through the increased number of water molecules in the solvation shell of sodium (Figure 1f, Figures S6 and S7).

### Electrocatalytic Performance of the Sodium Formate Concentration Series

First, we evaluated the electrolyte series of Na­(HCOO)·y H_2_O (with y = 4, 5, 8, 16, 55, 555) to probe how increasing formate concentrations affect the FOR activity on polycrystalline Pt, using cyclic voltammetry (CV) in a rotating disk electrode (RDE) setup (Figure 2). The conductivity values of the electrolytes listed in Table 1 range from 9 mS/cm to 124 mS/cm for Na­(HCOO)·555 H_2_O and Na­(HCOO)·8 H_2_O solutions, respectively. During FOR, ohmic drop compensation is used to account for the conductivity differences of the electrolytes.

**Figure 2:**
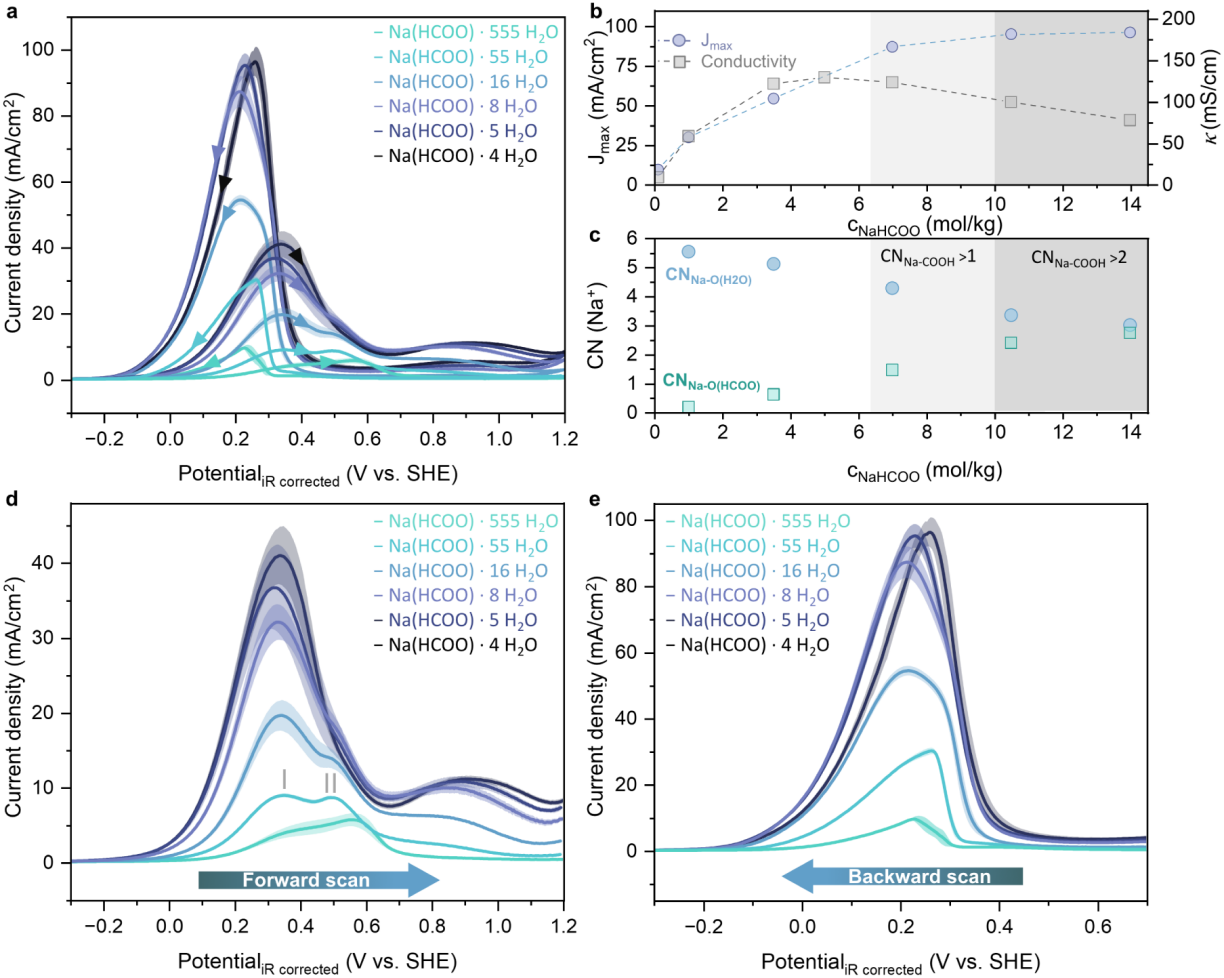
(a) Current response during cyclic voltammetry (50 mV/s) of the formate electrolyte series measured in a rotating disk electrode setup (1600 rpm) on polycrystalline Pt. The translucent area represents the standard deviation from at least three separate RDE measurements. (b) Variation of the current density peak maximum of the backward scan (J_max_) with the concentration of formate in the respective electrolyte (purple dots) and conductivity values of the formate electrolyte series (gray squares). (c) RDF-derived coordination number of sodium ions with water molecules (blue dots) and formate anions (green squares) of the formate electrolytes. (d) Forward scan of the cyclic voltammetry measurement depicted in (a). (e) Backward scan of the cyclic voltammetry measurement depicted in (a).

Figure 2a shows that, typical for FOR on polycrystalline Pt, the currents in the forward scan are lower than those in the backward scan for all concentrations studied.^5,22,23,41^ This behavior is explained by the dual pathway mechanism for FOR on Pt:^19,23,42,43^ the direct pathway involves formate oxidation directly to CO_2_ *via* a weakly adsorbed formate intermediate (eq 1), while adsorbed CO is formed first in the indirect pathway (eq 2), which is then subsequently oxidized to CO_2_ (eq 3). In the forward scan, adsorbed CO accumulates and poisons the catalyst surface, inhibiting the direct formate oxidation pathway, as the CO oxidation is slow and only takes place at high voltages (0.5–0.7 V vs the reversible hydrogen electrode (RHE)).^25,44^ Therefore, the FOR current response is diminished in the forward scan. In contrast, the backward scan starts at high potentials where adsorbed CO is removed, thus the current is predominantly related to the direct formate oxidation and is a good measure of the system’s catalytic activity for this pathway.^23,42^ During FOR, formate consumption is charge-compensated by hydroxide formation *via* hydrogen evolution at the Pt counter electrode (Supplementary Note 1, Figure S8).

1 $$HCOO^{-}\to CO_{2}+H^{+}+2e^{-}$$

2 $$HCOO^{-}+H^{+}\to CO_{ad}+H_{2}O$$

3 $$CO_{ad}+H_{2}O\to CO_{2}+2H^{+}+2e^{-}$$

Figure 2b shows that the current density maximum of the backward scan increases with formate concentration, reaching a plateau with current densities around 90–100 mA/cm^2^ in Na­(HCOO)·5 H_2_O–Na­(HCOO)·4 H_2_O. These current densities are 4-times higher than for the formic acid electrolyte of the same concentration (HCOOH·4 H_2_O, Figure S9). These findings align with previous,^19,29,42^ experimental and computational studies on Pt(111), which demonstrated that an increasing formate concentration, rather than formic acid concentration, enhances the direct FOR currents in 0.01–2 M HCOOH electrolytes. This results from adsorbed formate being currently recognized as the active species in FOR.^19,29,42^

To quantitatively investigate the concentration dependence, Figure S10 presents a double logarithmic plot of the current densities observed during the backward scan as a function of the formate concentration. If the formate concentration is directly proportional to the current density (and negligible coverage of adsorbed CO and bridge-bonded formate are assumed at the onset of the backward scan), a slope **b** = 1 should be observed $\left(j=Cons\tan t\times {[HCOO^{-}]}^{1}\right)$ .^29^ Our results show a slope of **b** = 0.97 at E = 0.36 V and **b** = 0.77 at E = 0.34 V. While this indicates that the formate concentration strongly influences current densities, it also suggests that additional factors are at play. As potentials approach the FOR peak maximum, the slope and the fit quality decrease, suggesting the stronger influence of other effects at higher current densities such as surface site availability or mass transport of formate. Indeed, for concentrations above 7 m NaHCOO (Na­(HCOO)·8 H_2_O), the peak maximum is nearly independent of the formate concentration (Figure 2a). This correlates well with the kinetic model by Salamon et al., which suggests that the surface site availability controls the FOR rate at the peak maximum of FOR,^29^ while the dynamic availability of formate dictates the overall current densities reached. In this context, dynamic formate availability describes the time-dependent concentration of reactive formate at the electrode–electrolyte interface, which can deviate from the bulk concentration, whereas surface site availability refers to unoccupied platinum sites accessible for formate oxidation.

Changes in electrolyte structure within the Na­(HCOO) electrolyte series can influence the FOR performance by altering (1) the dynamic availability of formate at the electrode surface, governed by its mass transport and local pH, and (2) surface site availability through CO adsorption and pH-dependent processes such as the OH^–^ adsorption and Pt oxidation.

We showed that increasing the formate concentration leads to increased Na^+^–HCOO^–^ ion pair and cluster formation, particularly in the WIS regime of the kosmotropic formate electrolytes (Figure 1). The increase in current density maximum (J_max_) with concentration from Na­(HCOO)·555 H_2_O to Na­(HCOO)·8 H_2_O is followed by its stagnation (Na­(HCOO)·8 H_2_O–Na­(HCOO)·4 H_2_O) and correlates with the initial increase (Na­(HCOO)·555 H_2_O → Na(HCOO)·16 H_2_O) as well as subsequent decrease (Na­(HCOO)·8 H_2_O → Na­(HCOO)·4 H_2_O) in the conductivity of the electrolytes (Figure 2b). The conductivity drop at high concentrations can be explained by increased ion pairing and clustering, as these larger, partially charge-neutralized aggregates are more sluggish.^45,46^ As such, a sharp decrease is observed when Na^+^–HCOO^–^ coordination number (CN) exceeds 1, indicating a direct relationship between ion cluster formation and poor transport properties (Figure 2b,c). This relationship is further demonstrated when comparing the conductivity of the pure formate WIS system to the ClO_4_-rich WIS system (Table 1): The Na­[(HCOO)_0.25_(ClO_4_)_0.75_]·4 H_2_O electrolyte with CN_Na–HCOO_ = 0.7 shows a nearly two-times higher conductivity than the Na­(HCOO)·4 H_2_O electrolyte with dominating Na^+^–HCOO^–^ ion aggregates (CN_Na–HCOO_ = 2.7). Although NaClO_4_ solutions in general show a slightly higher conductivity than NaHCOO (21% for1 m solutions), the significantly lower conductivity observed in NaHCOO-based WIS systems suggests that it is Na^+^–HCOO^–^ clustering which strongly hinders formate transport.

The viscosity of the electrolytes is also rising with increasing salt concentrations (Figure S11). However, the ClO_4_-rich electrolytes show an overall higher viscosity than the formate electrolytes (of equal molality) despite of their equal or higher conductivity, thus suggesting the dominant influence of ion clustering over viscosity on ion transport in the studied electrolyte series. The effect of viscosity and macroscopic mass transport limitations on J_max_ becomes more pronounced without RDE rotation (Figure S12): J_max_ in Na­(HCOO)·4 H_2_O is lower than J_max_ in Na­(HCOO)·5 H_2_O, correlating with its higher viscosity and lower self-diffusion coefficient of formate (D_HCOO_–, Table S2). Upon applying rotation, the impact of viscosity on J_max_ decreases, and varying rotations from 500 to 5000 rpm had no notable impact on the J_max_ magnitude, with only a peak potential shift observed for 500 rpm due to interfacial acidification in the Na­(HCOO)·4 H_2_O electrolyte (Figure S13). These findings indicate that formate transport through the ion-crowded double layer rather than bulk mass transport is the limiting factor. This structural electrolyte effect limits further increase in dynamic availability of formate at the interface and therefore explains the J_max_ plateau for the formate concentrations Na­(HCOO)·8 H_2_O–Na­(HCOO)·4 H_2_O (Figure 2b), even though the formate concentration in the electrolyte is increasing. Here we note, that while surface site availability is also likely decreasing, it is not the key factor behind the current density plateau, since reducing Na^+^–HCOO^–^ clustering with perchlorate addition enables further increase in current density (Figure 4a, *vide infra*).

Changes of the interfacial pH can play an important role, since formate anions can buffer only within mildly acidic conditions, when equilibrium concentrations of formic acid are present.^47^ Previous studies demonstrated a bell-shaped relationship between pH and FOR current^23,48^ in buffering supporting electrolytes. In contrast, in nonbuffering electrolytes, varying a bulk pH between 5 to 10 has little to no impact on the FOR current.^5,41,42^ Calculations in nonbuffering electrolytes show, that the interfacial pH remains nearly constant at 4, regardless of the bulk electrolyte pH.^42^ This interfacial acidification is a result of proton generation during formate oxidation (eq 1), while mass transport of formate and protons limits a further interfacial pH decrease.^42^ The interfacial pH window around 4–5 is associated with maximized FOR currents.^23,42^ At lower pH, this window is limited by the formate–formic acid equilibrium, whereas competitive OH-adsorption or Pt oxidation onset takes place at pH_interfacial_ > 6, thus decreasing currents.^23,42^ Here we note that due to the altered proton and water activity in high-concentration electrolytes,^6,15^ the optimal interfacial pH window can in principle shift. However, the underlying boundary processes are expected to remain valid, assuming that no change in the reaction mechanism is taking place.

To gain qualitative insights into the interfacial pH, we employed *in situ* surface-enhanced FTIR spectroscopy (SEIRAS) during CV measurements (Figure 3). Since the HCOO^–^/HCOOH ratio (pK_a_ ≈ 3.75, Supplementary Note 1, Table S3) strongly depends on the pH, characteristic carbonyl vibrations of formic acid (1709 cm^–1^) and formate (1581 cm^–1^) can be utilized for a qualitative estimation of the interfacial pH change during CV (Figure S14, Figure 3). SEIRAS spectra during formate oxidation in Na­(HCOO)·55 H_2_O (Figure 3a) and Na­(HCOO)·4 H_2_O (Figure 3b) show a rising vibration band at 1709 cm^–1^ assigned to formic acid while the formate band at 1581 cm^–1^ is decreasing. The presence of formic acid indicates that the proton activity in Na­(HCOO)·4 H_2_O equals a pH_interfacial_ < 5.0 in dilute conditions since the equilibrium formic acid concentration at higher pH values is <5% and within the detection limit of our FTIR instrument (Figure S15).^23^ The observed interfacial acidification is in line with previously calculated interfacial pH values for nonbuffering electrolytes with similar bulk pH.^5,41,42^ Although precise quantitative determination was not feasible due to the simultaneous buffering and reactant role of formate, CV data suggests that a proton activity similar to pH_interfacial_ < 4 is unlikely: according to the prior works at pH < 4,^23,42^ the position of the FOR peak maximum (backward scan) would be shifted to higher potentials (0.4 V vs the standard hydrogen electrode (SHE)), contrary to the observed peak maxima in the 0.2–0.25 V vs SHE range (backward scan) here. Thus, the interfacial pH can be assumed to be between 4 and 5 during the FOR current peak maxima in electrolytes with concentrations ≥1 m NaHCOO. This pH range aligns closely with previously reported optimal conditions for achieving maximum FOR current densities in dilute electrolytes and matches our experimental observations of high FOR current densities in the backward scans.^5,23,42,49^

**Figure 3:**
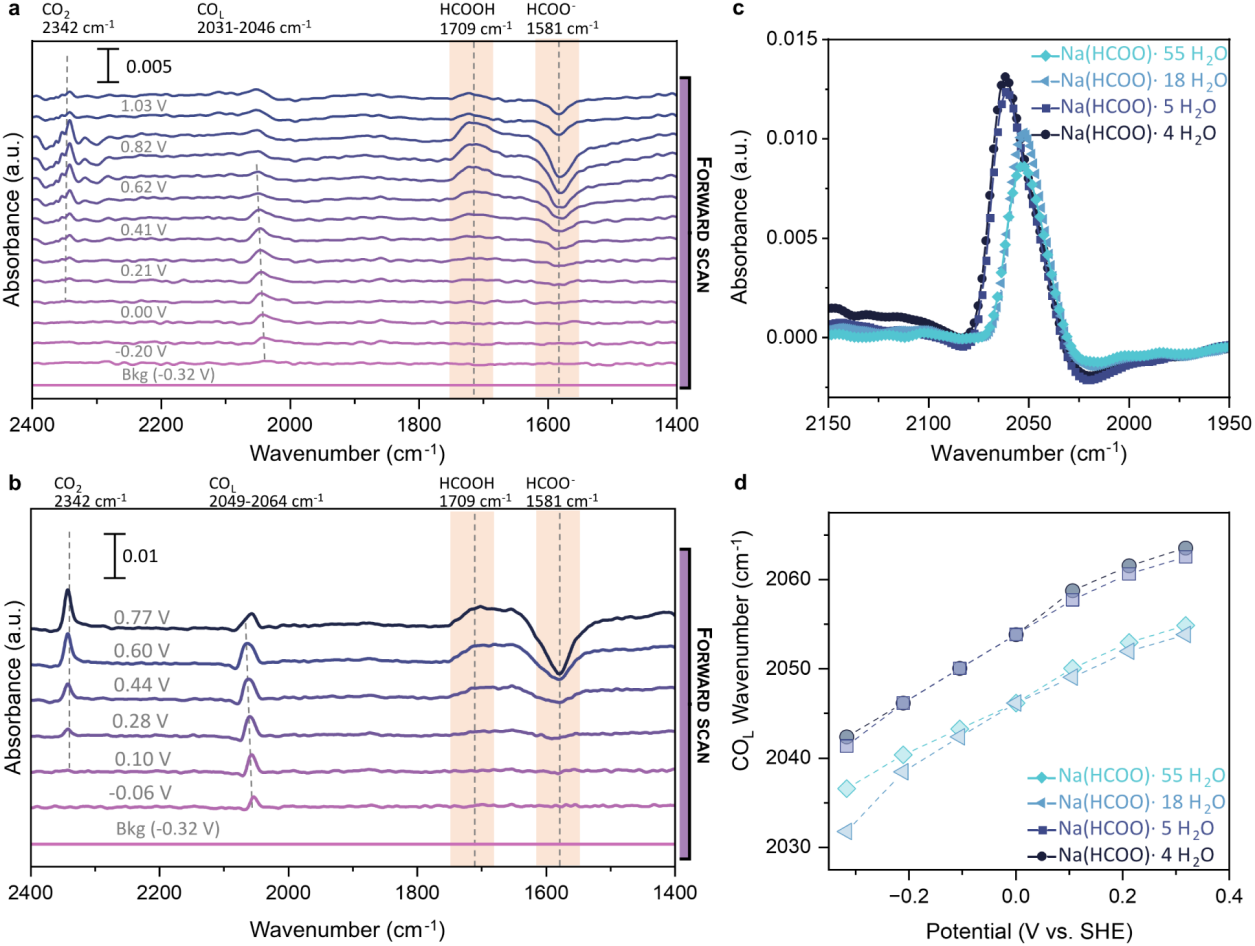
*In situ* SEIRAS spectra during formate oxidation in different formate electrolytes. (a) SEIRAS spectra during cyclic voltammetry from −0.3 to 1.2 V vs SHE (50 mV/s) in Na­(HCOO)·55 H_2_O; a spectrum was acquired every 2.14 s. (b) SEIRAS spectra during cyclic voltammetry from −0.3 to 0.8 V vs SHE (100 mV/s) in Na­(HCOO)·4 H_2_O; a spectrum was acquired every 1.68 s. (c) shows the linear CO adsorption band at 0.3 V during the forward scan (50 mV/s) of FOR. The evolution of the CO_L_ peak position as a function of the potential is depicted in (d). The background spectrum for all samples in (d) was obtained during the open circuit potential (OCV) (−0.19 V) of the Na­(HCOO)·55 H_2_O sample to compare the change in CO_L_ relative to Na­(HCOO)·55 H_2_O. The spectra of (a–b) and (c–d) were measured on the same SEIRAS coating to ensure similar enhancement conditions.

The CO poisoning on Pt is the main reason for decreased current densities in the forward scan of FOR.^23^ Interestingly, we observe an increase in current densities in the CV forward scan with formate concentration, especially in peak I, which can be primarily attributed to the direct formate oxidation and a minor contribution of CO preoxidation (Figure 2d).^48,50^ Peak II, previously assigned to the oxidation of CO,^23,48,50^ shows only a minor increase for electrolytes ≥Na­(HCOO)·18 H_2_O, suggesting comparable CO coverages in the high-concentration electrolytes. This is in contrast to formic acid electrolytes, for which the CO poisoning significantly increases with concentration, leading to diminished currents in the backward scan.^26^

To gain further insights, we used SEIRAS to compare the CO coverage in the electrolytes (Figure 3c, Figures S16–S17). Here we note that linear-bonded CO (CO_L_) is observed already for the background spectra collected at −0.3 V, due to the presence of the CO_ads_ within this potential range.^25^ Although oxidation currents are still small in this potential region, even low CO coverages can be readily detected owing to the high infrared cross section of the CO stretching mode.^51^ We reveal that the CO coverage of linear-bonded CO (CO_L_) increases with formate concentration, eventually reaching a plateau in the salt-solvate and WIS regimes (Na­(HCOO)·55 H_2_O < Na­(HCOO)·18 H_2_O < Na­(HCOO)·5 H_2_O ∼ Na­(HCOO)·4 H_2_O). The increase in CO_L_ coverage is visible through the increased CO peak intensity and blueshift to higher wavenumbers (2052 cm^–1^ to 2061 cm^–1^, Figure 3c and d). However, compared to the reported CO_L_ adsorption SEIRAS spectra of Okamoto et al. in concentrated formic acid (2089–2112 cm^–1^), the position of the CO_L_ adsorption band in WIS formate electrolytes is significantly lower (2061 cm^–1^), supporting a lower CO poisoning degree in the high-concentration formate electrolytes compared to formic acid as a lower CO band position is related to decreased interactions of neighboring CO molecules.^26^ The position and intensity of the observed CO_L_ band position of 2030–2065 cm^–1^ closely resemble those reported for low concentration NaHCOO (20 mM), suggesting that formate concentration is not the primary driving force behind CO poisoning.^48^

Bridge-bonded CO (CO_B_) was also observed during FOR, but the differences between electrolytes were more difficult to resolve due to background changes (Figure S18). Stark shifts for the CO_L_ band position are within the range of 30–37 cm^–1^ for all investigated electrolytes, indicating similar electronic environments of the CO molecules (Figure 3d) and are in agreement with CO oxidation studies in dilute electrolytes.^52^ The observed similarity in local electronic environments of the CO molecules is in line with the preserved water structure in the kosmotropic formate WIS electrolyte.

### Comparison of Formate Electrolytes with Perchlorate WIS Systems

Next, to decouple FOR performance differences from the increase in formate concentration and a modified water structure in WIS systems, we examined the [Na(HCOO)_x_(ClO_4_)_1–x_]·4 H_2_O (x = 0.007, 0.07, 0.25, 0.5, 0.75) electrolyte series. Their FOR performance was compared to the corresponding pure formate electrolytes of equal formate molality.

Figure 4a reveals the impact of the electrolyte structure on FOR changes with perchlorate/formate ratio. When comparing the electrolytes with the lowest perchlorate/formate ratio [Na­(HCOO)_0.75_(ClO_4_)_0.25_]·4 H_2_O and Na­(HCOO)·5 H_2_O (both contain 10.5 m NaHCOO), the current response is similar but an increase in J_max_ current density is observed for the ClO_4_-containing electrolyte, surpassing J_max_ of the pure formate electrolyte series (Figure 4a). The equal formate concentration of the systems should lead to similar surface site availability in the backward scan, suggesting that the difference in J_max_ is instead related to an increased formate availability. The addition of chaotropic perchlorate reduces cluster formation for sodium formate (CN_Na–HCOO_ drops from 2.4 to 2.1 in the first coordination shell and from 5.6 to 4.6 in the second coordination shell with ClO_4_ addition in the [Na(HCOO)_0.75_(ClO_4_)_0.25_]·4 H_2_O system, Table S1), leading to an increase in the dynamic availability at the interface and consequently of J_max_. The reduced clustering is further reflected in the formate self-diffusion coefficient (D_HCOO_−) in [Na(HCOO)_0.75_(ClO_4_)_0.25_], which is comparable to Na­(HCOO)·5 H_2_O despite a higher viscosity of the perchlorate containing electrolyte (2.3·10^–6^ vs 2.2·10^–6^ cm^2^/s, Table S6), suggesting a change in nanoscale mass transport properties. A similar performance increase, although below J_max_ of [Na(HCOO)_0.75_(ClO_4_)_0.25_]·4 H_2_O, was observed upon triflate (OTf) addition ([Na­(HCOO)_0.75_(OTf)_0.25_]·4 H_2_O), which is also considered a chaotropic anion and is positioned right below perchlorate in the Hofmeister scale (Figure S19). The electrolytes [Na­(HCOO)_x_(ClO_4_)_1–x_]·4 H_2_O with x < 0.5 show no change in J_max_ compared to the pure formate electrolyte of equal molality, since at these relatively low formate concentrations, there is no significant presence of ion clusters that would limit J_max_ (Figure 4a). Given the similarity in J_max_ values within this concentration range, an effect of perchlorate addition on surface-site limitation is unlikely.

**Figure 4:**
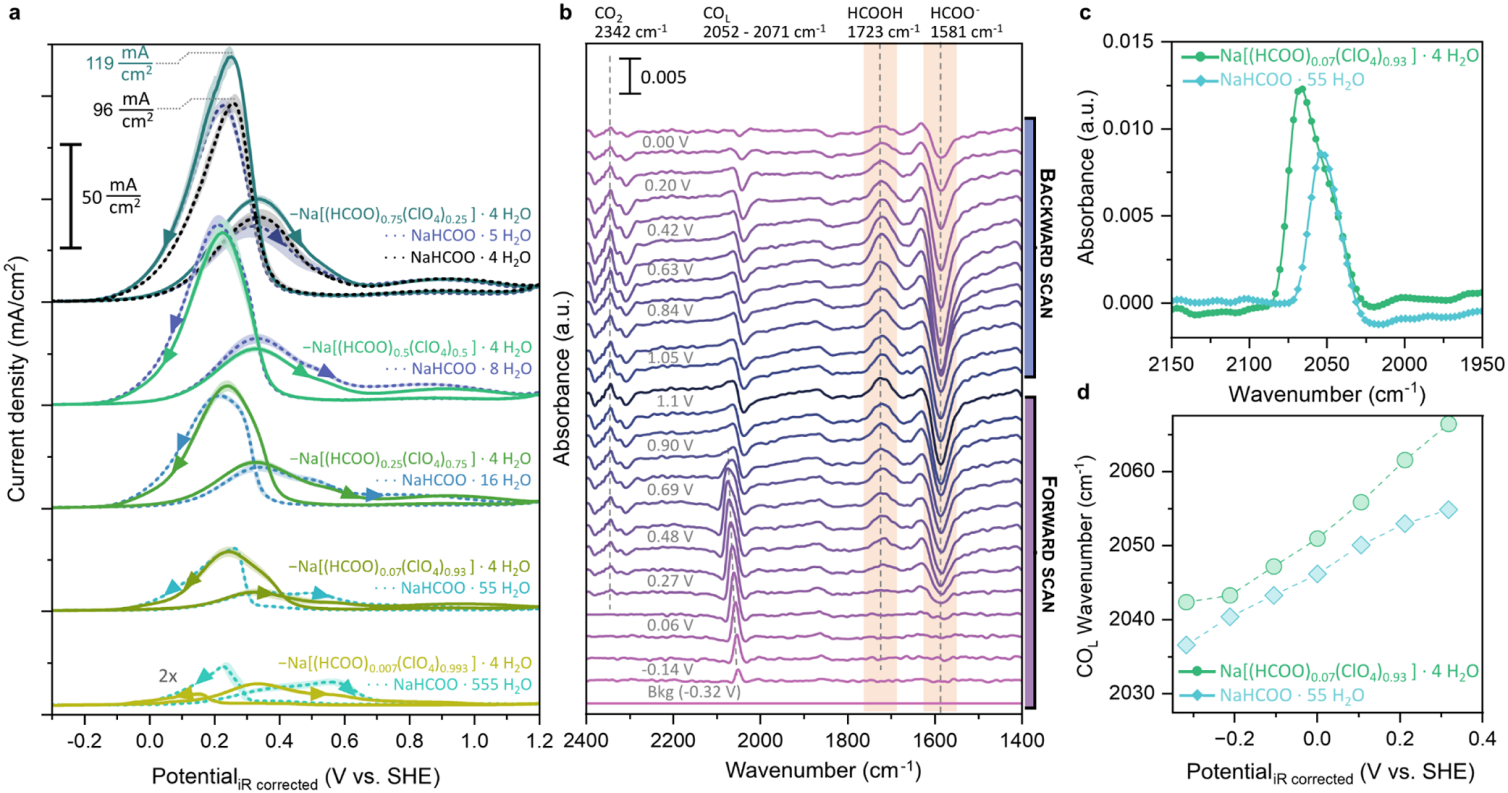
Electrochemical and spectroscopic study of the mixed perchlorate-formate electrolyte series. (a) Current response during cyclic voltammetry (50 mV/s) of the mixed formate-perchlorate electrolyte series and Na­(HCOO)·14 H_2_O measured in a rotating disk electrode setup (1600 rpm). The translucent area represents the standard deviation from at least three separate RDE measurements. For comparison, the cyclic voltammetry current response of the pure formate electrolyte with the same formate concentration is depicted for each perchlorate-formate system in dotted lines. (b) SEIRAS spectra during cyclic voltammetry from −0.3 to 1.2 V vs SHE (50 mV/s) in Na­[(HCOO)_0.07_(ClO_4_)_0.93_]·4 H_2_O; a spectrum was acquired every 2.14 s. (c) shows the linear CO adsorption band at 0.3 V during the forward scan (50 mV/s) of FOR. The evolution of the CO_L_ adsorption peak position is depicted in (d). The background spectrum of (c) and (d) was obtained during the OCV (−0.19 V) of the Na­(HCOO)·55 H_2_O sample to compare the change in CO_L_ in respect to the diluted formate electrolyte sample. The spectra of (c) and (d) were measured on the same SEIRAS coating to ensure similar enhancement conditions.

However, for Na­[(HCOO)_x_(ClO_4_)_1–x_]·4 H_2_O with x = 0.25 and 0.07, a clear shift of the formate oxidation onset toward higher potentials in the backward scan is observed compared to the respective NaHCOO electrolyte series. SEIRAS measurements in the Na­[(HCOO)_0.07_(ClO_4_)_0.93_]·4 H_2_O electrolyte (Figure 4b) revealed the presence of carbonyl vibrational band of HCOOH at 1723 cm^–1^, shifted to higher wavenumbers, due to the altered H-bonding of formic acid in the ClO_4_-containing WIS electrolytes.^53^ This band persists throughout the measurement and becomes particularly prominent during the backward scan, indicating sustained acidification of interfacial pH. This effect can be attributed to the hindered transport of protons in WIS electrolytes, due to H-bond disruption by perchlorate, and contrasts with Na(HCOO)·55 H_2_O electrolyte, where pH recovers between forward and backward scan, as observed from the decrease in HCOOH peak (Figure S20). In chaotropic WIS electrolytes, the hydrogen-bond network is strongly disrupted as the individual water domains are smaller and the fraction of isolated water molecules increases (Figure 1b). This altered electrolyte structure significantly impedes proton transport *via* the Grotthuss mechanism, as previously demonstrated.^54,55^ Because protons are formed during the formate oxidation reaction, their slower transport away from the interface leads to sustained interfacial acidification, as indicated by the larger and continuous presence of the formic acid band in the SEIRAS spectrum in Figure 4b, Figure S20. Additionally, the interfacial formate band at 1581 cm^–1^ shows a stronger decrease compared to Na­(HCOO)·55 H_2_O, consistent with protonation of formate to formic acid (Figure S20). Since the current densities reached in the electrolytes are similar or even smaller in the Na­[(HCOO)_0.07_(ClO_4_)_0.93_]·4 H_2_O electrolyte, these changes in the interfacial pH cannot be ascribed to differences in FOR currents (Figure 4a). In contrast, sustained interfacial acidification was not observed for the Na­[(HCOO)_0.75_(ClO_4_)_0.25_]·4 H_2_O electrolyte (Figure S21), demonstrating that the addition of small chaotropic perchlorate amounts does not result in notably impeded proton transport.

It was shown that the FOR reaction onset is highly pH dependent because it is related to the shift of Pt oxidation and OH^–^ adsorption with pH.^23^ Formate oxidation on an oxidized Pt surface is strongly hindered and competing OH^–^ adsorption further limits FOR currents. Since both processes are shifted to lower potentials with increasing pH, FOR currents decrease with rising interfacial pH.^23^ Furthermore, the WIS electrolyte structure itself can influence the oxidation and reduction behavior of Pt. Figure S22 shows a shift in Pt oxidation and reduction onset in 14 m NaClO_4_ electrolytes compared to 1 m NaClO_4_, leading to less pronounced Pt oxidation in the same potential window. These changes in Pt oxidation behavior could be attributed to the altered water activity and coverage, thereby influencing OH^–^ adsorption. Consequently, the continuous interfacial acidification due to highly disrupted water structure is beneficial for FOR, as it shifts Pt reduction in the backward scan to higher potentials and enables the earlier FOR reaction onset in [Na­(HCOO)_x_(ClO_4_)_1–x_]·4 H_2_O with x = 0.07 and 0.25.

For the WIS electrolyte with the lowest formate concentration ([Na­(HCOO)_0.007_(ClO_4_)_0.993_]·4 H_2_O), the currents in the backward scan are strongly diminished compared to the pure Na­(HCOO)·555 H_2_O electrolyte, and unlike typical FOR CV profiles, the current in the backward scan is lower than in the forward scan (Figure 4a). We propose that this is due to significantly limited dynamic availability of formate as a result of macroscopic mass transport limitations due to disrupted H-bonding in this electrolyte and higher electrolyte viscosity coupled with low formate concentration and surface acidification.

Differences are also observed in the forward scan for [Na­(HCOO)_0.07_(ClO_4_)_0.93_]·4 H_2_O, particularly in peak II, which is mainly related to the oxidation of adsorbed CO. SEIRAS spectra of the CO_L_ adsorption in [Na(HCOO)_0.07_(ClO_4_)_0.93_]·4 H_2_O (Figure 4c,d and Figure S23) reveal an increased CO_L_ adsorption peak and Stark shift compared to the pure formate electrolyte with the same molarity (Na­(HCOO)·55 H_2_O). This suggests changes in the electronic environment of the CO molecule either due to altered composition of the electrochemical double layer or reduced H-bond interactions as a result of lower water content. However, we note that the observed increased CO population *via* SEIRAS contradicts the decreased CO oxidation peak in the CV (Figure 4a), pointing to complex interactions between adsorbed CO and the altered electrolyte environment.

## Conclusions

This work establishes a mechanistic link between electrolyte structure and formate oxidation activity on Pt in concentrated and water-in-salt regimes (Figure 5). We identify a key trade-off: while increasing sodium formate concentration boosts the direct oxidation pathway by improving reactant availability, higher concentrations induce kosmotropic Na^+^–HCOO^–^ pairing and multi-ion clustering. Such clustering decreases conductivity and reactant transport, thus limiting further current increase. Introducing chaotropic anions, such as ClO_4_^–^, can mitigate this limitation by disrupting ion clustering and pushing the maximum formate oxidation current (J_max_) beyond the limit observed in pure-formate electrolytes. However, excess ClO_4_^–^ disrupts the water hydrogen-bonding network, hindering proton transport and decreasing the interfacial pH, as evidenced by SEIRAS.

**Figure 5:**
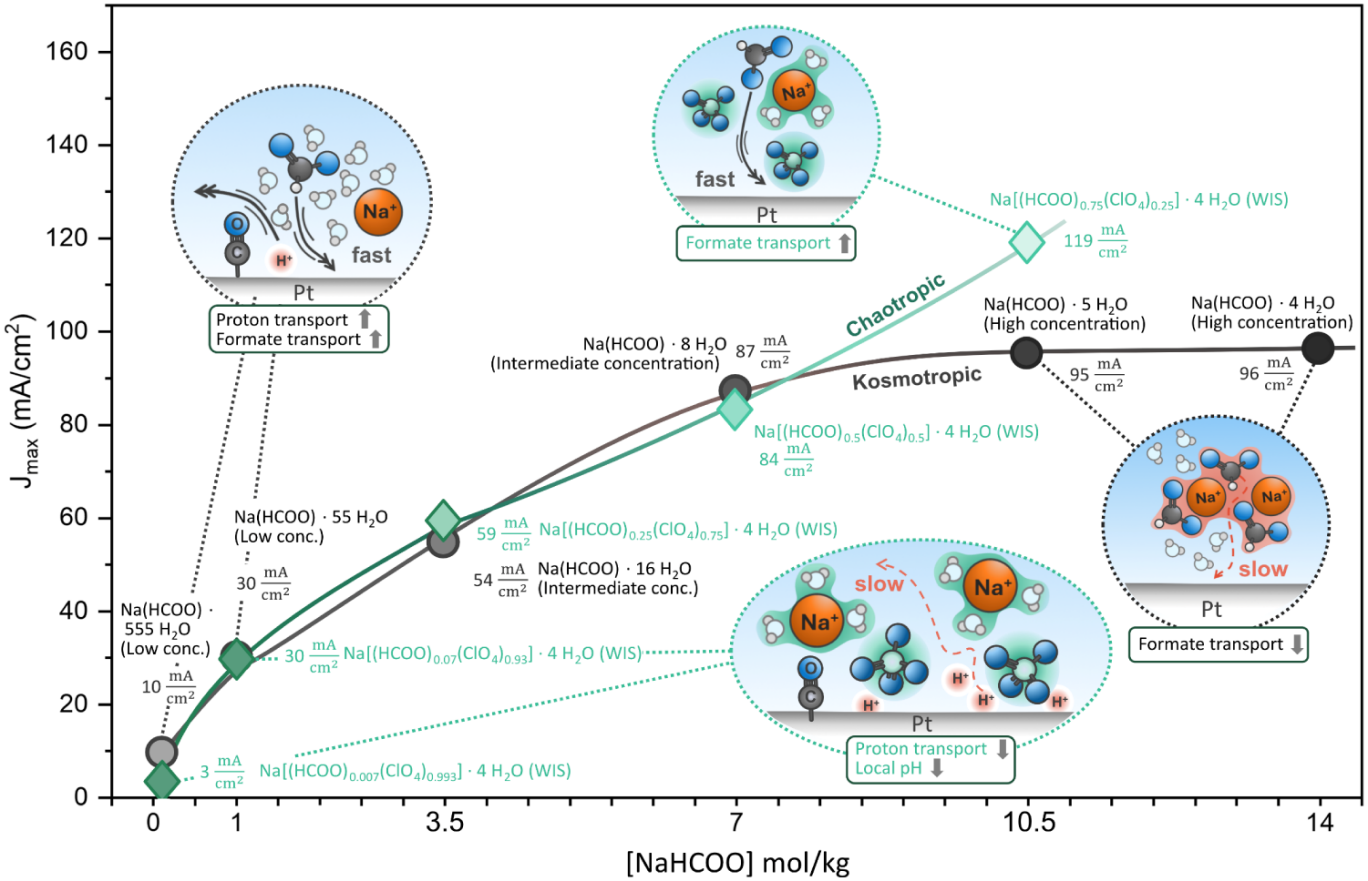
Schematic overview of the concentration-dependent variations in FOR current density maximum through the changed electrolyte composition and related interfacial processes.

Ultimately, we show that the optimization of concentrated electrolytes extends beyond simply increasing reactant concentration—it also involves managing structural trade-offs. For instance, introducing chaotropic additives can mitigate ion clustering to improve reactant transport, but their excess can simultaneously disrupt the hydrogen-bond network, which governs proton transport and poisoning. To achieve optimal catalytic performance, electrolytes must be tuned to balance ion–ion and ion–water interactions.

## Methods

### Electrochemical Methods

Electrolytes were prepared using high-purity salts (sodium formate: 99.998% Sigma-Aldrich, sodium perchlorate hydrate: 99.99% Sigma-Aldrich) and ultrapure water (18.2 MΩ, Millipore). The electrolytes were purged with Ar for 30 min before each experiment and kept under Ar atmosphere during the experiment. All cyclic voltammetry measurements (scanning rate of 50 mV/s) were conducted in a rotating disk electrode setup (RRDE-3A, ALS Japan) with a standard three-electrode electrochemical cell and a rotation speed of 1600 rpm unless otherwise stated. A platinum coil counter electrode and leakless Ag/AgCl reference were used to prevent Cl^–^ contamination. All glassware was cleaned prior experiments through repetitive boiling in nitric acid (10%) and ultrapure water. A polycrystalline Pt disk electrode (4 mm diameter) was used as the working electrode and was mirror-polished (diamond polish: 3 μm, 1 μm, and 0.25 μm) and sonicated in water before each measurement (Figure S24). The potential was controlled using a BioLogic VSP-300 potentiostat. In-situ IR compensation of 85% was applied during the measurements and the open circuit potential of each electrolyte was determined before cyclic voltammetry measurements (Figure S25, Table S4). Potentials were converted from the Ag/AgCl reference scale to the SHE scale *via* $E_{SHE}=E_{\left(Ag/AgCl,3.4M\;KCl\right)}^{0}+E_{\left(Ag/AgCl,3.4M\;KCl\right)}$ . The potential of each leakless Ag/AgCl reference electrode (EDAQ) was frequently verified and remained within $E_{\left(Ag/AgCl,3.4M\;KCl\right)}^{0}=205\;mV\pm 20\;mV$ vs SHE. The potential was plotted vs SHE instead of the pH-corrected RHE scale as the pH of highly concentrated electrolytes is challenging to determine precisely. The accuracy of pH probes is limited due to an increased (unknown) liquid junction potential and decreasing water activity. The obtained pH values for highly concentrated electrolytes in Table S5 should be therefore considered as approximation.

### Electrolyte Characterization

*Ex situ* FTIR spectra of the electrolytes were measured in ATR configuration using a Nicolet iS50 FTIR instrument equipped with a Veemax III ATR unit and a Si wafer (IRUBIS) as ATR element. Each spectrum consists of 128 coadded scans and was acquired with a resolution of 4 cm^–1^ using a high-resolution MCT detector. Nuclear magnetic resonance (NMR) spectra were recorded on a Bruker ASCEND 400 spectrometer. ^1^H spectra were acquired in a double walled NMR tube with the D_2_O reference in the separated compartment, to avoid shifts of the D_2_O reference peak appearing in high-concentration solutions. Diffusion-ordered spectroscopy (DOSY) NMR experiments were performed utilizing double-walled NMR tubes and the standard dstebpgp3s pulse sequence. The array of gradient strength covered 2.408–45.74 G/cm and was linearly divided into 16 steps. The diffusion delay and gradient pulse duration for each sample are denoted in Table S6. The diffusion coefficients were calculated by fitting the peak integrals according to the Stejskal–Tanner equation (Figure S26). Conductivity was obtained *via* the Oakton PC2700 benchtop meter after calibration of the sensor with standard solutions (Oakton). pH measurements were conducted utilizing the Mettler Toledo InLab Max Pro-ISM pH probe and each electrolyte was measured four times to determine the average and standard deviations.

### In-Situ FTIR Spectroscopy

SEIRAS-active Pt thin films were prepared on Si wafers (IRUBIS, specialized 1) through a two-step procedure previously published: Briefly, Au was deposited *via* an electroless deposition procedure followed by the electrochemical deposition of platinum.^56,57^ The resistance of the prepared thin films was generally <20 Ω. The complete coverage of the gold layer with Pt was confirmed *via* cyclic voltammetry in 0.1 M HClO_4_, showing the typical features of Platinum (Figure S27), as well as scanning electron microscopy (SEM, Hitachi S-4800 microscope at 2 kV acceleration voltage), which shows the nanoscale-rough Pt surface consisting of closely packed Pt nanoparticles continuously covering the surface (Figure S28). The Pt thin film was electrochemically cleaned in 0.05 M HClO_4_ through repeated cycling and the roughness of the film was determined as described previously (Figure S27). The FTIR measurements were conducted in a home-built single-compartment spectroelectrochemical cell equipped with a Pt coil counter electrode and a leakless Ag/AgCl reference electrode (EDAQ) as depicted in Figure S29. To avoid accumulation of bubbles at the working electrode, the electrolyte was circulated through the cell with a peristaltic pump (15 mL/min). The electrochemical cell was situated on a nitrogen-purged Veemaxx III with an angle of incidence of 35°. The spectra were acquired with a resolution of 8 cm^–1^ with a high-resolution MCT detector unless otherwise stated. The potential was controlled with a BioLogic VSP-300 potentiostat and IR compensation of 85% was applied during the measurements (Figure S30). The open circuit spectrum of Na­(HCOO)·55 H_2_O was chosen as the background for all spectra in Figures 3c and 4c, to track changes in the CO adsorption amount in relation to the Na­(HCOO)·55 H_2_O electrolyte, assuming that this sample will have the lowest CO coverage. For this measurement series, all acquisitions were conducted on the same Pt thin film and 0.35 V vs SHE was chosen as upper potential limit to avoid changes of the Pt thin film structure at higher voltages and to ensure similar conditions and surface-enhancement for all electrolytes. Similarly, the *in situ* spectra depicted in Figure 3a,b and Figure 4b were also measured on the same Pt thin film to ensure comparability between electrolytes. The background of those spectra was taken within the first 2 s of the CV, beginning at −0.32 V. To facilitate direct comparison of the interfacial acidification in the electrolytes, the scan rate of NaHCOO·4 H_2_O was adjusted to 100 mV/s to ensure a comparable total charge passed enabling the detection of mass transport effects without the influence of increased proton production (Figure S31).

### Molecular Dynamics Simulation

Classical molecular dynamics (MD) simulations were carried out using the LAMMPS program package (version 23 Jun 2022).^58^ The compositions and equilibrated box dimensions of the simulated systems are listed in Table S7. The SPC/E force field^59^ was used for water. OPLS-AA^60^ parameters were used for Na^+^, and OPLS-2009IL^61^ parameters were used for HCOO^–^ and ClO_4_^–^. Geometric mixing rules were applied for nonbonded interactions between different atom kinds. The cutoff distance for both Coulombic and Lennard-Jones interactions was set to 1.2 nm. The time step was set to 0.5 fs. Initial configurations for all simulations were generated by random placement within the simulation box using PACKMOL.^62^ The Nosé–Hoover^63,64^ chain thermostat and barostat were employed to maintain an average pressure of 1.01325 bar in all NpT simulations and a temperature of 298.15 K in all NpT and NVT simulations. To avoid energetic hotspots, the simulation protocol began with an initial energy minimization followed by a 0.03 ns NVE run with added velocity scaling corresponding to a temperature of 500 K. The system was then cooled down to the production temperature of 298.15 K and equilibrated in the NpT ensemble for 4 ns. The simulation box length was fixed to its average value over the latter 2 ns, after which the system was further equilibrated for 2 ns in the NVT ensemble. Finally, a 15 ns production run was performed in the NVT ensemble, with the trajectory saved at an interval of 500 fs. Radial distribution function and neighbor count analyses were performed with the postprocessing code TRAVIS.^65^

## Supplementary Material



## Notes

Additional experimental details and methods; NMR measurements; SEIRAS setup and spectra; calculation parameters; complementary RDE measurements (PDF)

## #

International Center for Young Scientists (ICYS), National Institute for Materials Science, 1-1 Namiki, Tsukuba, Ibaraki 305-0044, Japan
