# Supplementary material for: Electrolyte Structure Governs Formate Oxidation in Water-in-Salt Systems
Source: J Am Chem Soc. 2026 Mar 19;148(12):12672–83. doi: 10.1021/jacs.5c19298 (PMC13047694; doi:10.1021/jacs.5c19298)
Supplement: Supplementary file 1 [file ja5c19298_si_001.pdf]

Supplementary information

Electrolyte Structure Governs Formate Oxidation in Water-in-Salt Systems

Katharina Trapp[a], Soracha Kosasang[a,b], Johannes Ingenmey[c,d], Dario Gomez Vazquez[a], Manuel Reiter[a] Mathieu Salanne[c,d,e], Maria R. Lukatskaya[a,*]

[a]Electrochemical Energy Systems Laboratory, Department of Mechanical and Process Engineering, ETH Zurich, 8092 Zurich, Switzerland

[b]Present address: International Center for Young Scientists (ICYS), National Institute for Materials Science, 1-1 Namiki, Tsukuba, Ibaraki 305-0044, Japan

[c]Réseau sur le Stockage Electrochimique de l'Energie (RS2E), CNRS FR3459, 80039 Amiens, France

[d]Sorbonne Université, CNRS, Physicochimie des Électrolytes et Nanosystemes Interfaciaux, PHENIX, F-75005 Paris, France

[e]Institut Universitaire de France (IUF), 75231 Paris, France

* Corresponding author email: mlukatskaya@ethz.ch

Table of Contents

Supplementary Figures 5

Figure S1. ^{13}C-NMR spectra of selected electrolytes from the formate electrolyte series. Depicted is the NMR-signal of the carbonyl carbon atom of the formate anion........ 5

Figure S2. MD generated radial distribution function (rdf) of the pure sodium formate electrolytes for the intermolecular interactions (a, c) and coordination numbers (b, d) of sodium with the oxygen atoms of water and the oxygen atoms of formate. 5

Figure S3. MD generated rdf in the WIS formate/perchlorate electrolytes for the intermolecular interactions (a, c) and coordination numbers (b, d) of sodium with the oxygen atoms of water and the oxygen atoms of the formate anion........ 6

Figure S4. MD generated rdf in the WIS formate/perchlorate electrolytes for the intermolecular interactions (a) and coordination numbers (b) of sodium with the oxygen atoms of ClO_4^-. 6

Figure S5. FTIR spectra of the Cl-O vibration of the ClO_4^- anion. With increasing sodium perchlorate concentration, the peak redshifts from 1103 cm^{-1} ($Na[(HCOO)_{0.75}(ClO_4)_{0.25}] \cdot 4\ H_2O$) to 1086 cm^{-1} ($NaClO_4 \cdot 4H_2O$)........ 7

Figure S6. MD simulation box of (a) $NaHCOO \cdot 55\ H_2O$, (b) $NaHCOO \cdot 16\ H_2O$, (c) $NaHCOO \cdot 8\ H_2O$, (d) $NaHCOO \cdot 5\ H_2O$. The colored balls of yellow, blue, white and black represent Na^+, oxygen, hydrogen and carbon, respectively. 7

Figure S7. MD simulation box of (a) $Na[(HCOO)_{0.5}(ClO_4)_{0.5}] \cdot 4\ H_2O$, (b) $Na[(HCOO)_{0.75}(ClO_4)_{0.25}] \cdot 4\ H_2O$. The colored balls of yellow, blue, white, green and black represent Na^+, oxygen, hydrogen, chloride and carbon, respectively. 8

Figure S8. Pt counter electrode potential during RDE experiments in dilute and high-concentration formate electrolytes (a). ^{1}H-NMR before and after 50 formate oxidation CV cycles in 10.5 m NaHCOO (b). ^{13}C-NMR before and after 50 formate oxidation CV cycles in 10.5 m NaHCOO (c). 8

Figure S9. Cyclic voltammetry (50 mV/s) of sodium formate (a) and formic acid (b) electrolytes with different concentrations........ 8

Figure S10. Double logarithmic plot of current density vs. formate concentration at a defined potential during the onset of the backward scan of the cyclic voltammetry. If the current density is proportional to the formate concentration, a slope of one would be expected. 9

Figure S11. Viscosity of $NaHCOO \cdot y\ H_2O$ and $Na[(HCOO)_x(ClO_4)_{1-x}] \cdot 4\ H_2O$ electrolytes........ 9

Figure S12. Cyclic voltammetry (50 mV/s) of $NaHCOO \cdot 4\ H_2O$, $NaHCOO \cdot 5\ H_2O$ and $Na[(HCOO)_{0.75}(ClO_4)_{0.25}] \cdot 4\ H_2O$ at 0 rpm rotation rate. 10

Figure S13. Cyclic voltammetry (50 mV/s) in 14 m NaHCOO at different RDE rotation speeds. At 500 rpm, the potential corresponding to J_{max} is shifted to higher values due interfacial acidification arising from insufficient macroscopic mass transport of protons at this rotation rate. To enhance the effect of rotation rate on interfacial acidification, the upper limit of the cyclic voltammetry was set to 0.9 V, thus facilitating a stronger interfacial acidification by reducing time between forward and backward scan. The presence of HCOOH features in SEIRAS spectra (Figure 3) confirms this local pH drop, which shifts the oxidation potential. 10

Figure S14. FTIR spectra of formic acid and sodium formate solutions with different concentrations. Depicted is the carbonyl vibration region to show the band position of the C-O vibration in formic acid and formate. 11

Figure S15. Determination of the formic acid detection limit of our IR setup based on its vibrational band at 1708 cm^{-1}. The background spectrum was taken in 1 m sodium formate electrolyte and then slowly

acidified using formic acid, while monitoring the pH. The measurements were conducted in the same electrochemical cell as the *in situ* measurements. The spectra were offset for clarity.................................11

Figure S16. CO peak maximum intensity derived from *in situ* FTIR spectra as a function of potential for Na(HCOO) · y H_2O with y= 55,18,5,4 (a) and Na[(HCOO)$_{0.25}$(ClO_4)$_{0.75}$] · 4 H_2O, Na(HCOO) · 55 H_2O (b)........12

Figure S17. *In situ* SEIRAS spectra of the CO_L region in different formate electrolytes during linear sweep voltammetry (50mV/s) from -0.32 to 0.32V. A spectrum was taken every 100 mV. 12

Figure S18. SEIRAS spectra of the CO region during linear sweep voltammetry (a,b). Marked is the CO_L and CO_B adsorption region. The CO bridge adsorption region is difficult to interpret with rising salt concentration due to artefacts from background substraction, related to the large difference in salt concentration between the background and sample spectrum. The OCV spectrum of 1 m NaHCOO was used as the background for all spectra to ensure comparable conditions in terms of CO_B intensity. Additionally, all spectra were measured on the same SEIRAS thin film electrode to ensure similar surface-enhancement...13

Figure S19. Cyclic voltammetry (50 mV/s) of NaHCOO · 4 H_2O, Na[(HCOO)$_{0.75}$(ClO_4)$_{0.25}$] · 4 H_2O and Na[(HCOO)$_{0.75}$(OTf)$_{0.25}$] · 4 H_2O. The electrolytes containing the chaotropic anions triflate and perchlorate surpass J_{max} of NaHCOO · 4 H_2O, which shows the highest performance in the NaHCOO · y H_2O electrolyte series. The observed peak shift in both forward and backward scan for Na[(HCOO)$_{0.75}$(OTf)$_{0.25}$] indicates differences in interfacial pH compared to the perchlorate system. This might be related to the difference in water activity, ion interactions or impurities in the sodium triflate electrolyte...13

Figure S20. Forward and backward scan *in situ* SEIRAS spectra during cyclic voltammetry from -0.3 V to 1.2 V vs. SHE (50 mV/s) in Na(HCOO) · 55 H_2O (a) and Na[(HCOO)$_{0.07}$(ClO_4)$_{0.93}$] · 4 H_2O (b); a spectrum was acquired every 2.14s. The background of (a-b) was taken within the first 2s of each CV, beginning at -0.32 V. 14

Figure S21. Forward and backward scan of *in situ* SEIRAS spectra during cyclic voltammetry from -0.32 V to 0.45 V vs. SHE (50 mV/s) in Na[(HCOO)$_{0.25}$(ClO_4)$_{0.75}$] · 4 H_2O (a); a spectrum was acquired every 2.1s. The background spectrum is the first spectrum of the cyclic voltammetry measurement covering the voltage window -0.32 – 0.20 V. (b) current response during SEIRAS measurement in Na[(HCOO)$_{0.25}$(ClO_4)$_{0.75}$] · 4 H_2O. Passed total charge during the forward scan in Na[(HCOO)$_{0.25}$(ClO_4)$_{0.75}$] · 4 H_2O (c)...15

Figure S22. Cyclic voltammetry (100 mV/s) in diluted and WIS perchlorate electrolytes.The upper potential boundary is varied between 1.4 V vs. SHE (a) and 1.2 V vs. SHE (b) to evaluate the oxidation current of Platinum within the respective boundaries and electrolyte...15

Figure S23. *In situ* SEIRAS spectra of the CO_L region in Na[(HCOO)$_{0.07}$(ClO_4)$_{0.93}$] · 4 H_2O during linear sweep voltammetry (50mV/s) from -0.32 to 0.32 V. A spectrum was taken every 100 mV. 16

Figure S24. Cyclic voltammetry (500 mV/s) of the used polycrystalline Pt RDE electrode in 0.1 M $HClO_4$. The H-UPD region during cyclic voltammetry was used for the determination of the electrochemical active surface area (ECSA) as previously described. [3] The roughness factor of the RDE electrode was determined to be ~ 1.14...16

Figure S25. Monitored open circuit potential in each electrolyte before cyclic voltammetry measurements. 17

Figure S26. Linear DOSY NMR plot showing ln(peak intensity) as a function of the squared pulsed field gradient strength and the corresponding fit quality based on the Stejskal-Tanner equation (a-f)...............17

Figure S27. Cyclic voltammetry (50 mV/s) of the thin film Pt electrode used for the SEIRAS experiments in 0.05 M $HClO_4$ (a). The roughness factor of the Pt thin film was determined based on the H-UPD region in the CV, as previously reported.[3] Comparison of cyclic voltammetry response (50 mV/s) from incompletely deposited Pt thin films (b) with the cyclic voltammetry of the continuous Pt thin film depicted in (a)..18

Figure S28. Plan-view SEM pictures acquired at different magnifications from the as-prepared Pt thin film utilized for SEIRAS measurements. 18

Figure S29. *In situ* SEIRAS cell design. The electrolyte was pumped through the cell to ensure proper convection during the measurement. The pumping speed was limited to 15 ml/min to avoid film delamination of the working electrode. 19

Figure S30. Current response in SEIRAS cell during formate oxidation in $Na[(HCOO)_{0.07}(ClO_4)_{0.93}] \cdot 4\ H_2O$ (a), $NaHCOO \cdot 55\ H_2O$ (b) and $NaHCOO \cdot 4\ H_2O$ (c). The oscillations observed in the current response are typical for formate oxidation when far from equilibrium.[4] FOR peak maxima are shifted to higher potentials in the SEIRAS cell due to enhanced acidification arising from reduced convection, high currents and surface roughness compared to RDE measurements as well as a higher resistance in the SEIRAS cell (~10 Ω vs. ~1 Ω). 19

Figure S31. Measured current as a function of time during SEIRAS experiments of the respective electrolytes. 20

Supplementary tables....21

Table S1. MD-derived coordination numbers of sodium ions with formate, water and perchlorate. The coordination numbers were taken from the local minima of the rdf function. 21

Table S2. NMR-derived self-diffusion coefficient of formate (D_{HCOO^-}), peak current density (J_{max}) and Viscosity in selected electrolytes. 21

D_{HCOO^-}....21

Table S3. Calculated formate/formic acid ratio at different pH values based on the formular denoted in supplementary note 2. A pKa value of 3.75 was used for formic acid. 21

Table S4. Open circuit potential of the respective electrolytes. The last 10s of the OCV measurement vs. time were averaged for OCV determination, which show a standard deviation below 0.001 V. 22

Table S5. Measured pH values of the Ar-bubbled electrolytes. Each electrolyte was measured four times to determine average and standard deviation....22

Table S6. Selected gradient pulse duration and diffusion time for diffusion NMR experiments. 23

Table S7. Composition, equilibrated box length *L*, and density ρ_{MD} of the simulated systems....23

Supplementary notes 24

Supplementary note 1. Counter reaction and charge balancing of FOR in WIS electrolytes....24

Supplementary note 2: Calculation of formic acid/formate ratio with respect to the pH. 24

Supplementary references....25

Supplementary Figures

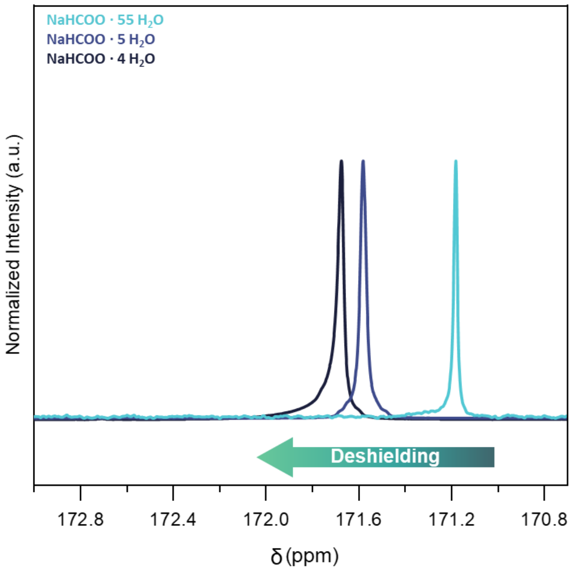

Figure S1. ^{13}C-NMR spectra of selected electrolytes from the formate electrolyte series. Depicted is the NMR-signal of the carbonyl carbon atom of the formate anion.

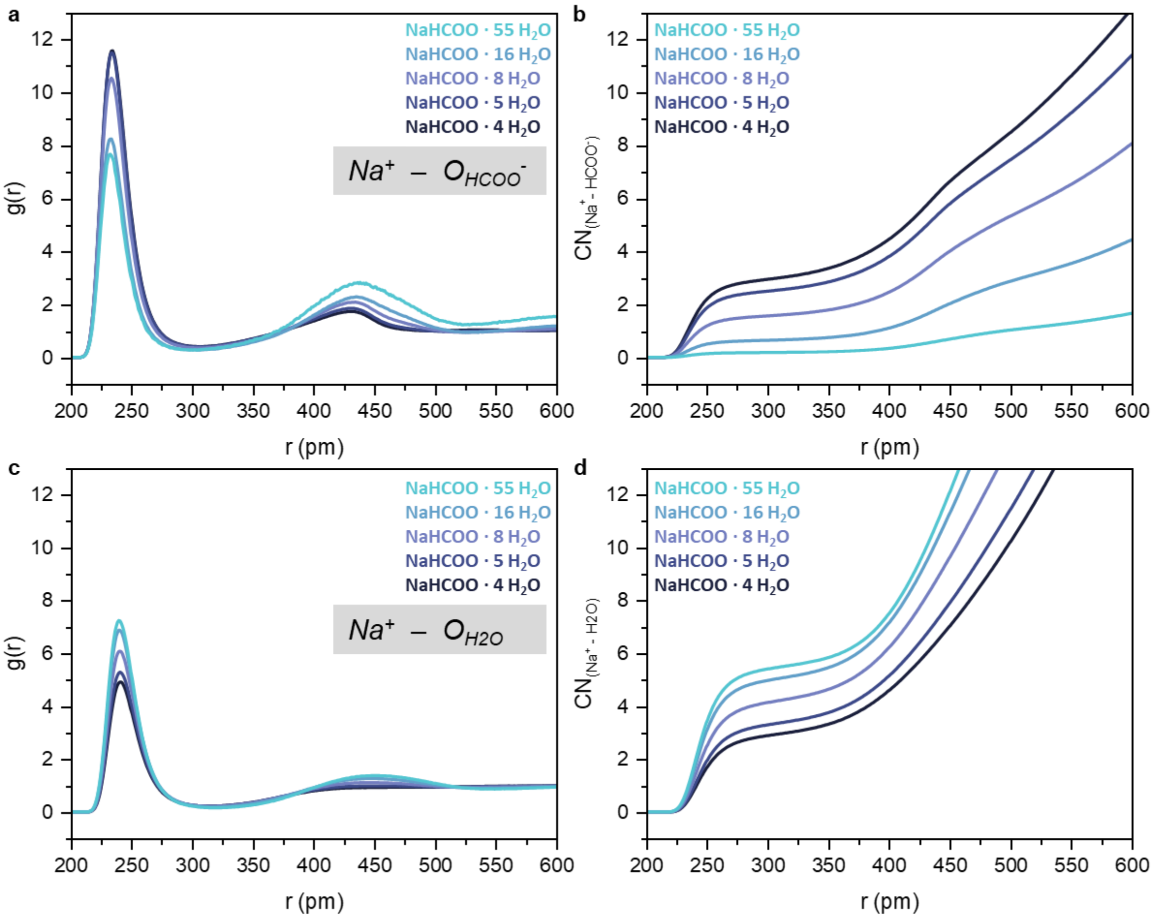

Figure S2. MD generated radial distribution function (rdf) of the pure sodium formate electrolytes for the intermolecular interactions (**a**, **c**) and coordination numbers (**b**, **d**) of sodium with the oxygen atoms of water and the oxygen atoms of formate.

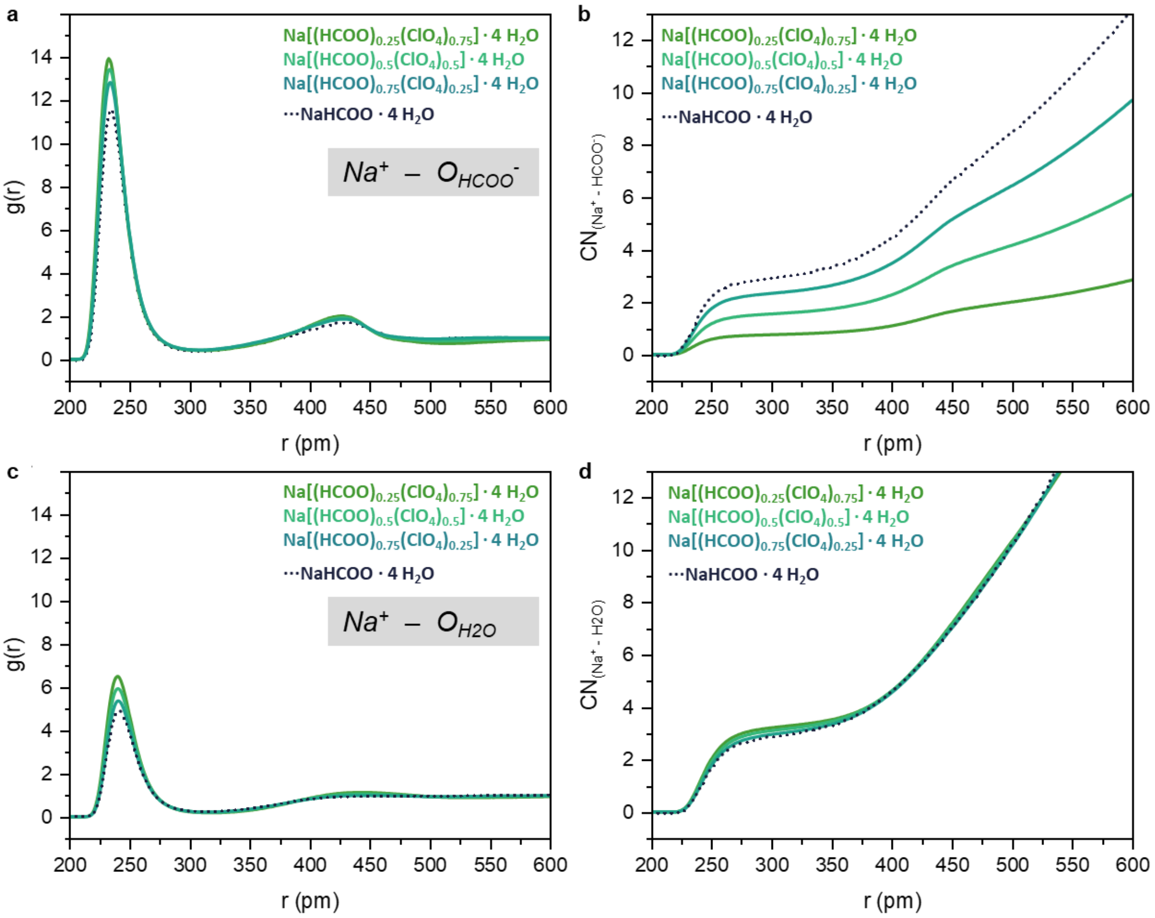

Figure S3. MD generated rdf in the WIS formate/perchlorate electrolytes for the intermolecular interactions (**a**, **c**) and coordination numbers (**b**, **d**) of sodium with the oxygen atoms of water and the oxygen atoms of the formate anion.

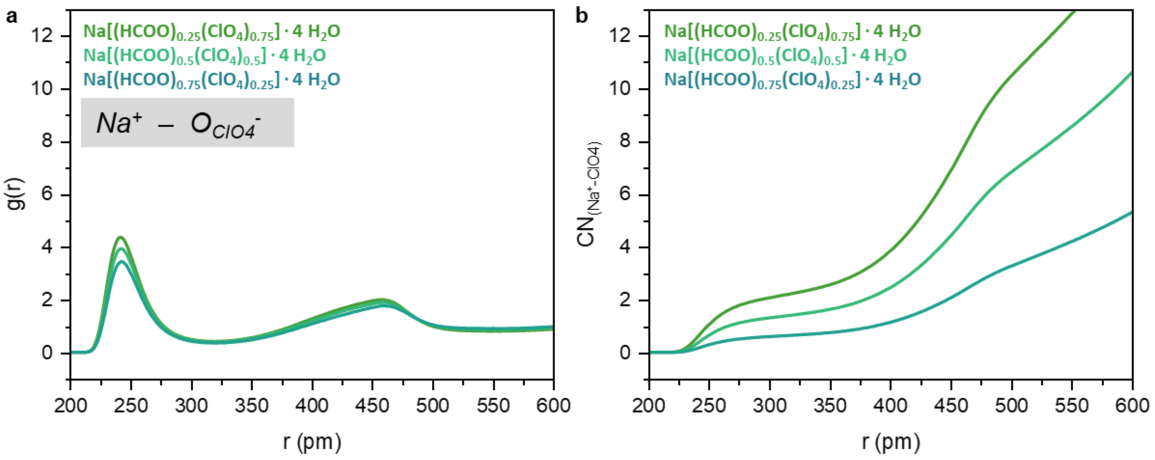

Figure S4. MD generated rdf in the WIS formate/perchlorate electrolytes for the intermolecular interactions (**a**) and coordination numbers (**b**) of sodium with the oxygen atoms of ClO_4^-.

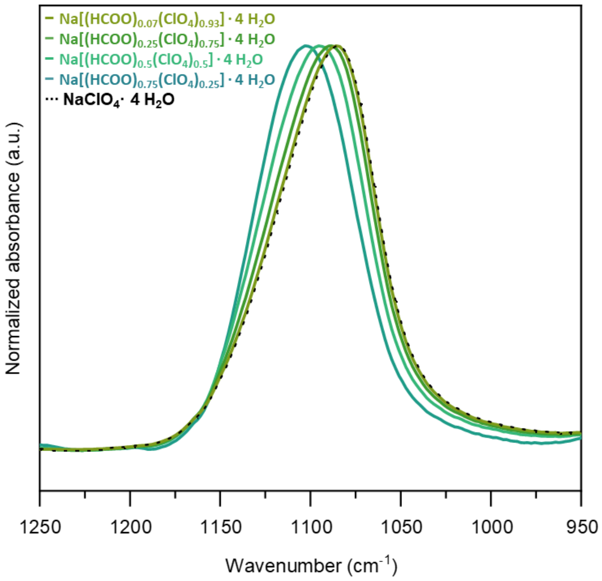

Figure S5. FTIR spectra of the Cl-O vibration of the ClO_4^- anion. With increasing sodium perchlorate concentration, the peak redshifts from 1103 cm^{-1} ($Na[(HCOO)_{0.75}(ClO_4)_{0.25}] \cdot 4\ H_2O$) to 1086 cm^{-1} ($NaClO_4 \cdot 4H_2O$).

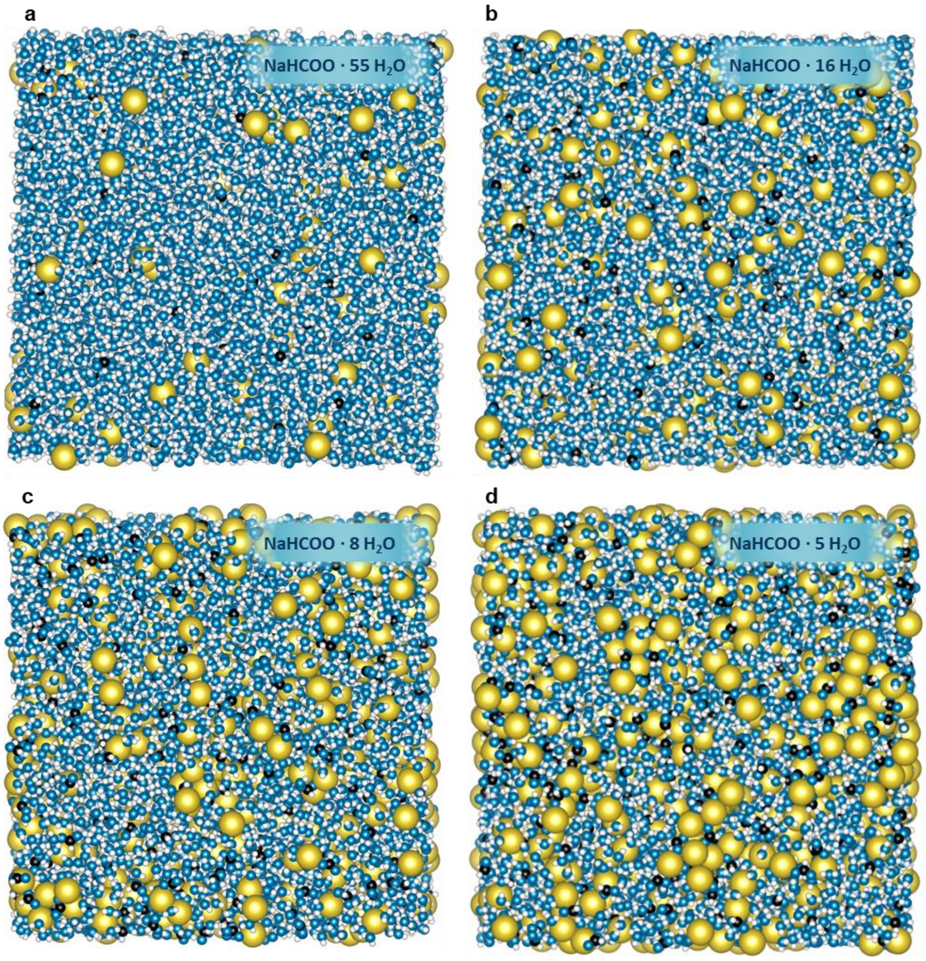

Figure S6. MD simulation box of (**a**) $NaHCOO \cdot 55\ H_2O$, (**b**) $NaHCOO \cdot 16\ H_2O$, (**c**) $NaHCOO \cdot 8\ H_2O$, (**d**) $NaHCOO \cdot 5\ H_2O$. The colored balls of yellow, blue, white and black represent Na^+, oxygen, hydrogen and carbon, respectively.

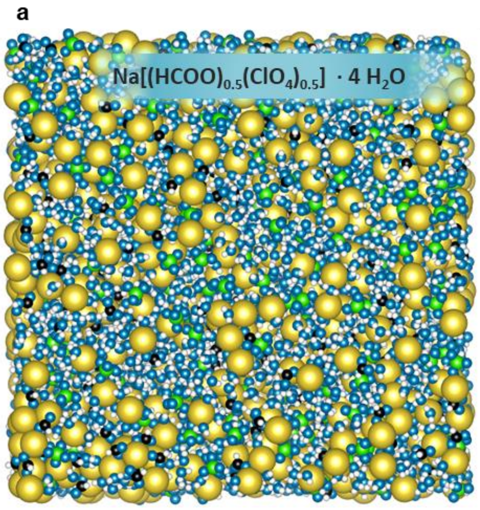

Figure S7. MD simulation box of (**a**) $Na[(HCOO)_{0.5}(ClO_4)_{0.5}] \cdot 4\ H_2O$, (**b**) $Na[(HCOO)_{0.75}(ClO_4)_{0.25}] \cdot 4\ H_2O$. The colored balls of yellow, blue, white, green and black represent Na^+, oxygen, hydrogen, chloride and carbon, respectively.

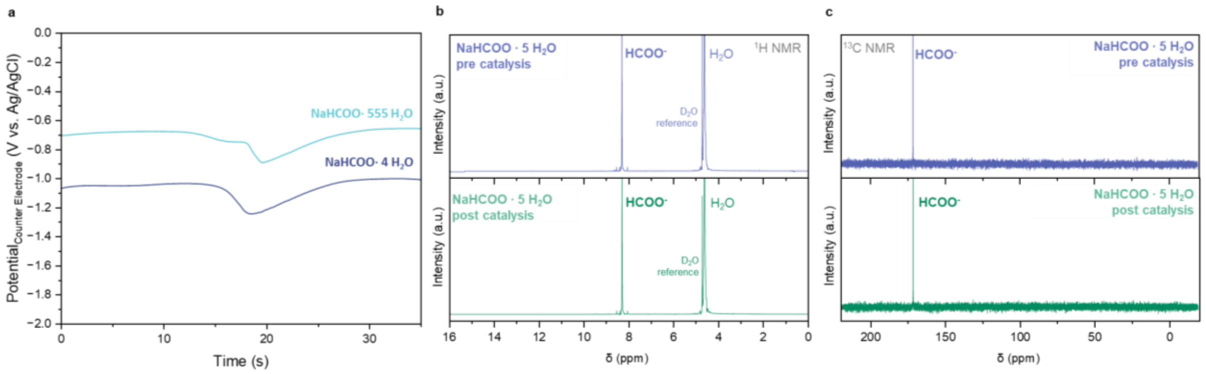

Figure S8. Pt counter electrode potential during RDE experiments in dilute and high-concentration formate electrolytes (**a**). ^{1}H-NMR before and after 50 formate oxidation CV cycles in 10.5 m NaHCOO (**b**). ^{13}C-NMR before and after 50 formate oxidation CV cycles in 10.5 m NaHCOO (**c**).

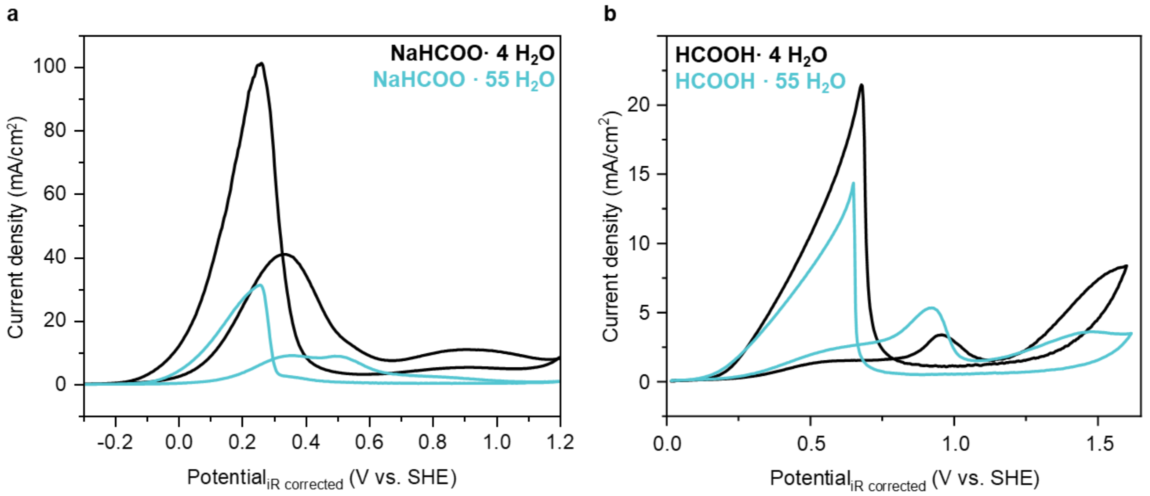

Figure S9. Cyclic voltammetry (50 mV/s) of sodium formate (**a**) and formic acid (**b**) electrolytes with different concentrations.

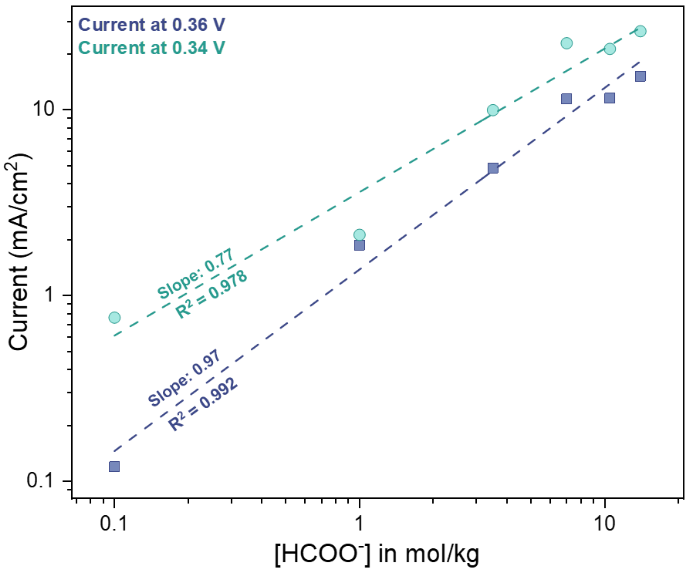

Figure S10. Double logarithmic plot of current density vs. formate concentration at a defined potential during the onset of the backward scan of the cyclic voltammetry. If the current density is proportional to the formate concentration, a slope of one would be expected.

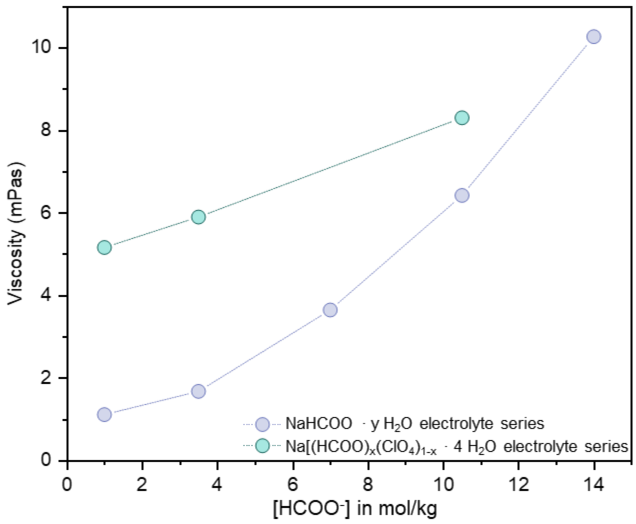

Figure S11. Viscosity of NaHCOO · y H_2O and Na[$(HCOO)_x(ClO_4)_{1-x}$] · 4 H_2O electrolytes.

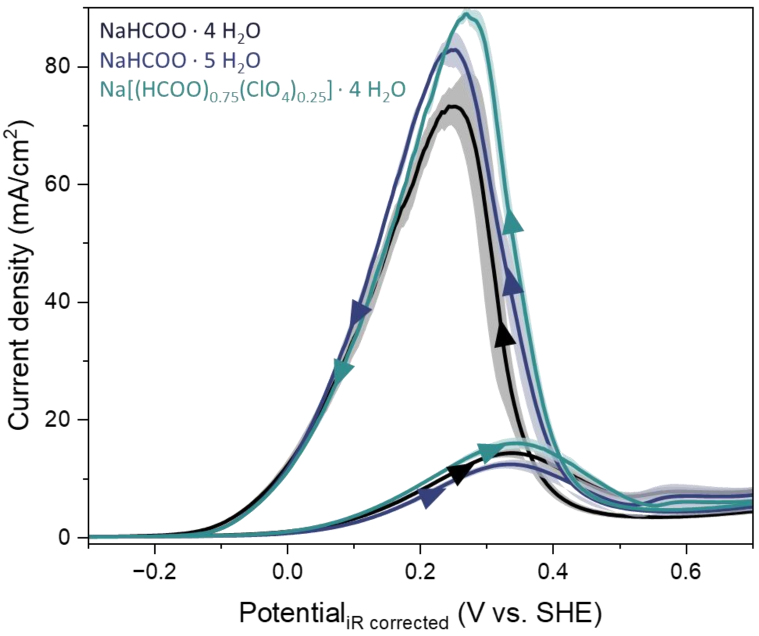

Figure S12. Cyclic voltammetry (50 mV/s) of NaHCOO · 4 H_2O, NaHCOO · 5 H_2O and Na[$(HCOO)_{0.75}(ClO_4)_{0.25}$] · 4 H_2O at 0 rpm rotation rate.

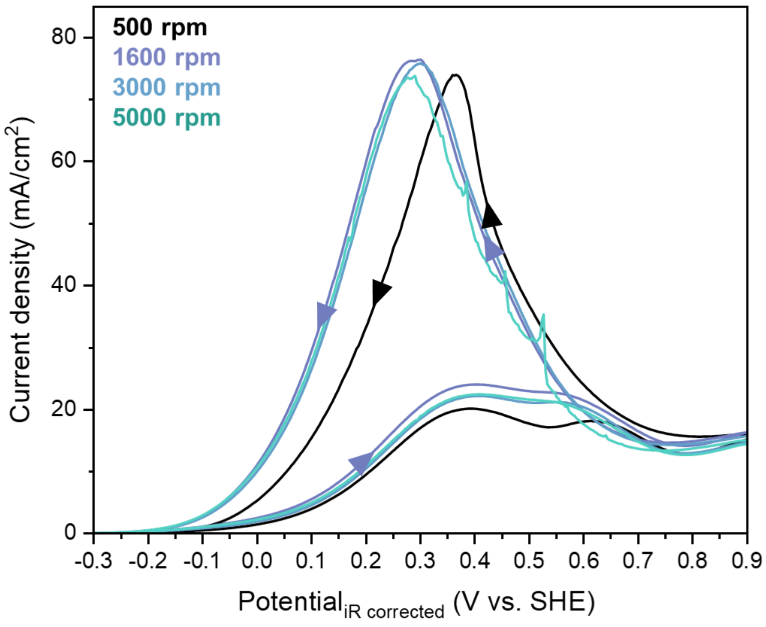

Figure S13. Cyclic voltammetry (50 mV/s) in 14 m NaHCOO at different RDE rotation speeds. At 500 rpm, the potential corresponding to J_{max} is shifted to higher values due interfacial acidification arising from insufficient macroscopic mass transport of protons at this rotation rate. To enhance the effect of rotation rate on interfacial acidification, the upper limit of the cyclic voltammetry was set to 0.9 V, thus facilitating a stronger interfacial acidification by reducing time between forward and backward scan. The presence of HCOOH features in SEIRAS spectra (**Figure 3**) confirms this local pH drop, which shifts the oxidation potential.

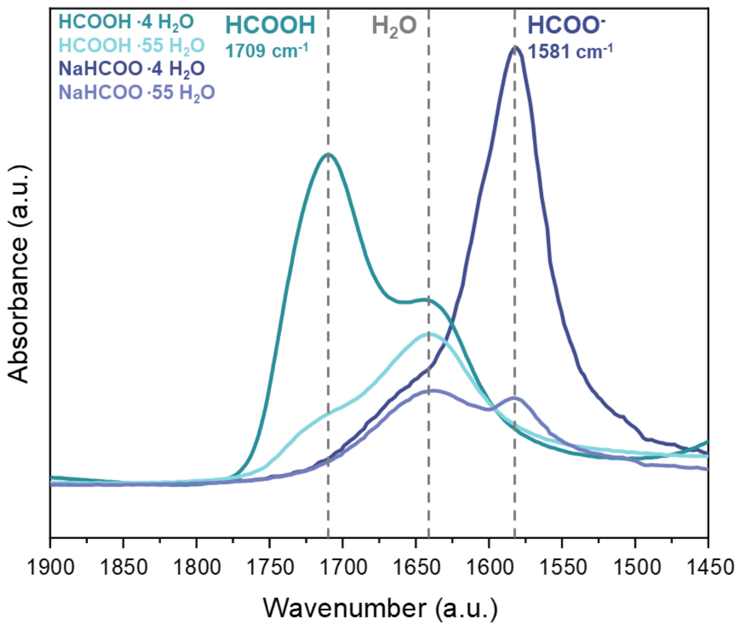

Figure S14. FTIR spectra of formic acid and sodium formate solutions with different concentrations. Depicted is the carbonyl vibration region to show the band position of the C-O vibration in formic acid and formate.

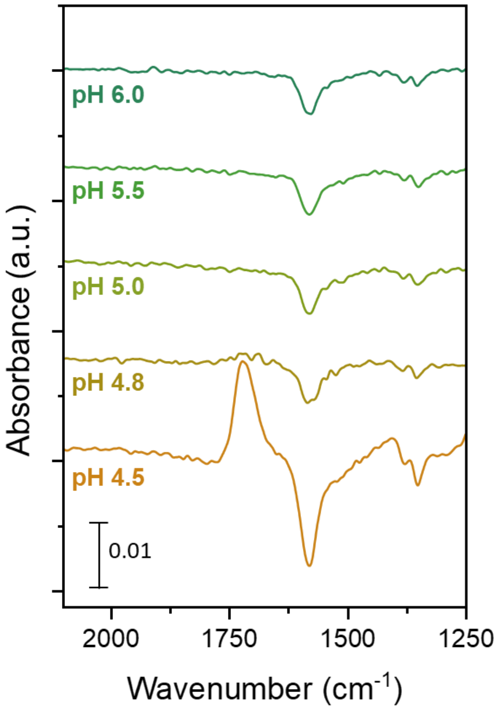

Figure S15. Determination of the formic acid detection limit of our IR setup based on its vibrational band at 1708 cm^{-1}. The background spectrum was taken in 1 m sodium formate electrolyte and then slowly acidified using formic acid, while monitoring the pH. The measurements were conducted in the same electrochemical cell as the *in situ* measurements. The spectra were offset for clarity.

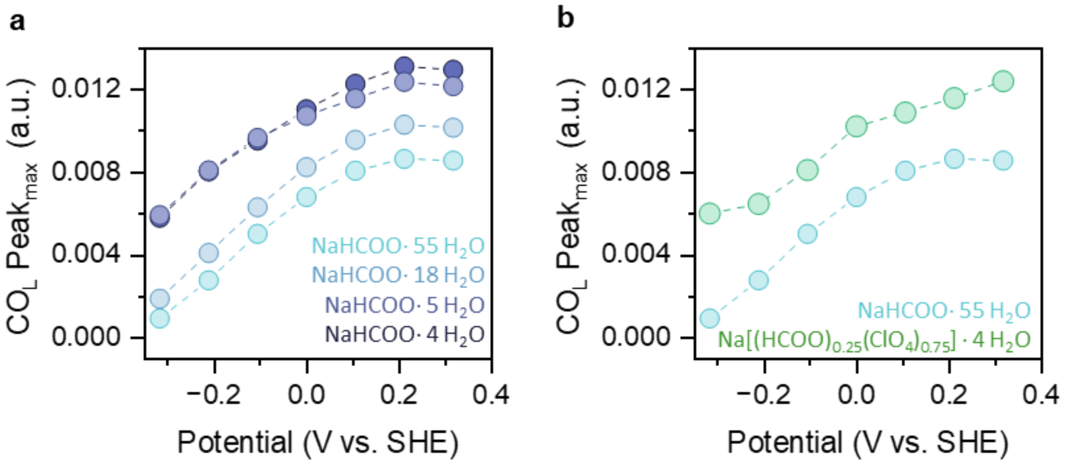

Figure S16. CO peak maximum intensity derived from *in situ* FTIR spectra as a function of potential for Na(HCOO) · y H_2O with y= 55,18,5,4 (**a**) and Na[(HCOO)$_{0.25}$(ClO$_4$)$_{0.75}$] · 4 H_2O, Na(HCOO) · 55 H_2O (**b**).

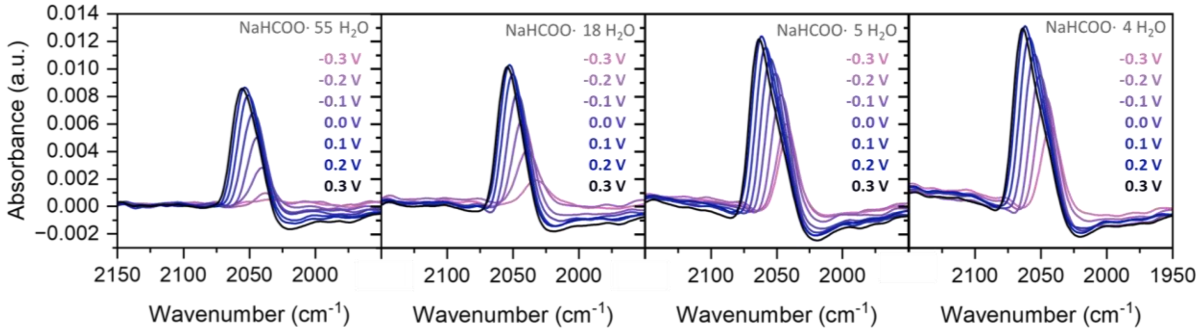

Figure S17. *In situ* SEIRAS spectra of the CO_L region in different formate electrolytes during linear sweep voltammetry (50mV/s) from -0.32 to 0.32V. A spectrum was taken every 100 mV.

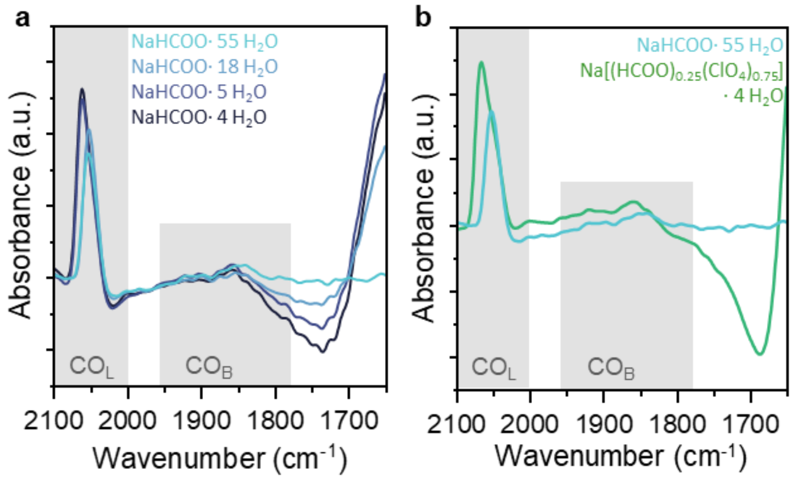

Figure S18. SEIRAS spectra of the CO region during linear sweep voltammetry (**a,b**). Marked is the CO_L and CO_B adsorption region. The CO bridge adsorption region is difficult to interpret with rising salt concentration due to artefacts from background substraction, related to the large difference in salt concentration between the background and sample spectrum. The OCV spectrum of 1 m NaHCOO was used as the background for all spectra to ensure comparable conditions in terms of CO_B intensity. Additionally, all spectra were measured on the same SEIRAS thin film electrode to ensure similar surface-enhancement.

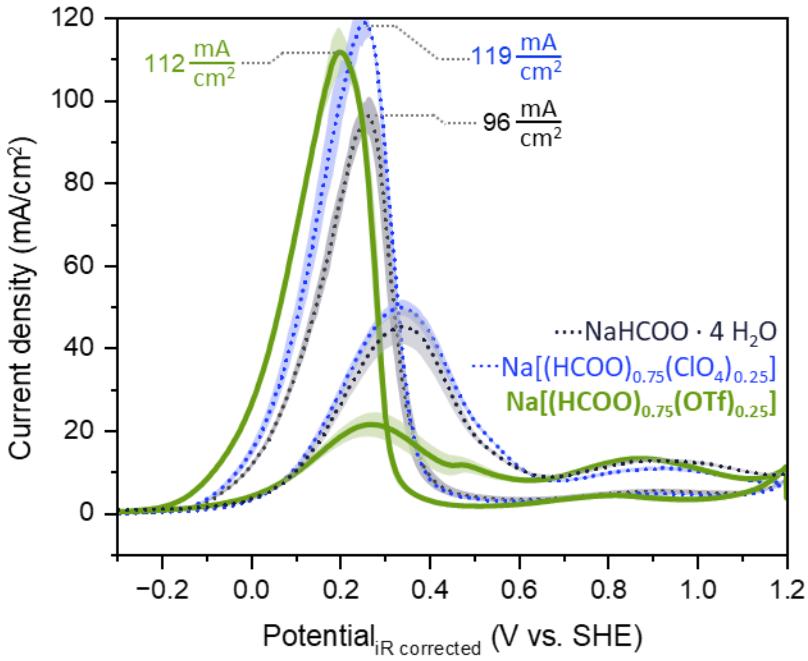

Figure S19. Cyclic voltammetry (50 mV/s) of NaHCOO · 4 H_2O, Na[$(HCOO)_{0.75}(ClO_4)_{0.25}$] · 4 H_2O and Na[$(HCOO)_{0.75}(OTf)_{0.25}$] · 4 H_2O. The electrolytes containing the chaotropic anions triflate and perchlorate surpass J_{max} of NaHCOO · 4 H_2O, which shows the highest performance in the NaHCOO · y H_2O electrolyte series. The observed peak shift in both forward and backward scan for Na[$(HCOO)_{0.75}(OTf)_{0.25}$] indicates differences in interfacial pH compared to the perchlorate system. This might be related to the difference in water activity, ion interactions or impurities in the sodium triflate electrolyte.

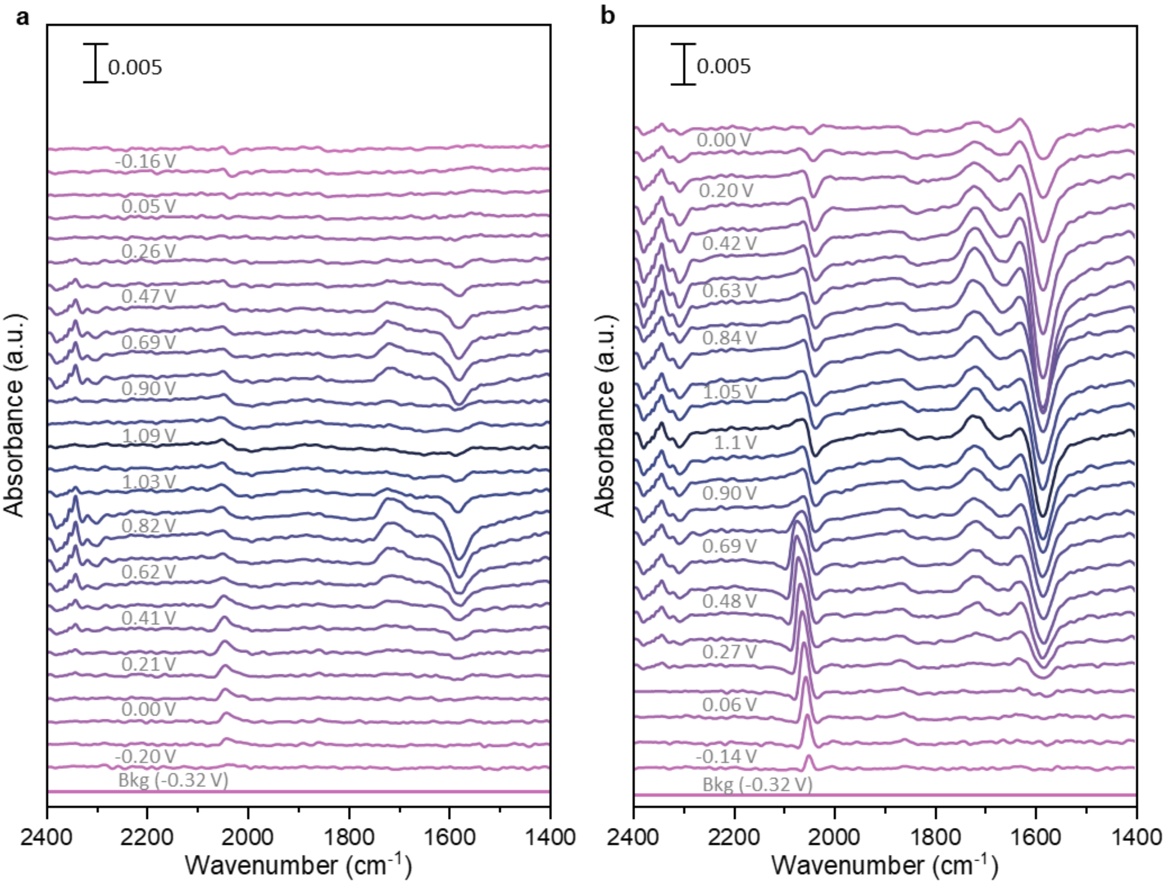

Figure S20. Forward and backward scan *in situ* SEIRAS spectra during cyclic voltammetry from -0.3 V to 1.2 V vs. SHE (50 mV/s) in Na(HCOO) · 55 H_2O (**a**) and Na[$(HCOO)_{0.07}(ClO_4)_{0.93}$] · 4 H_2O (**b**); a spectrum was acquired every 2.14s. The background of (**a-b**) was taken within the first 2s of each CV, beginning at -0.32 V.

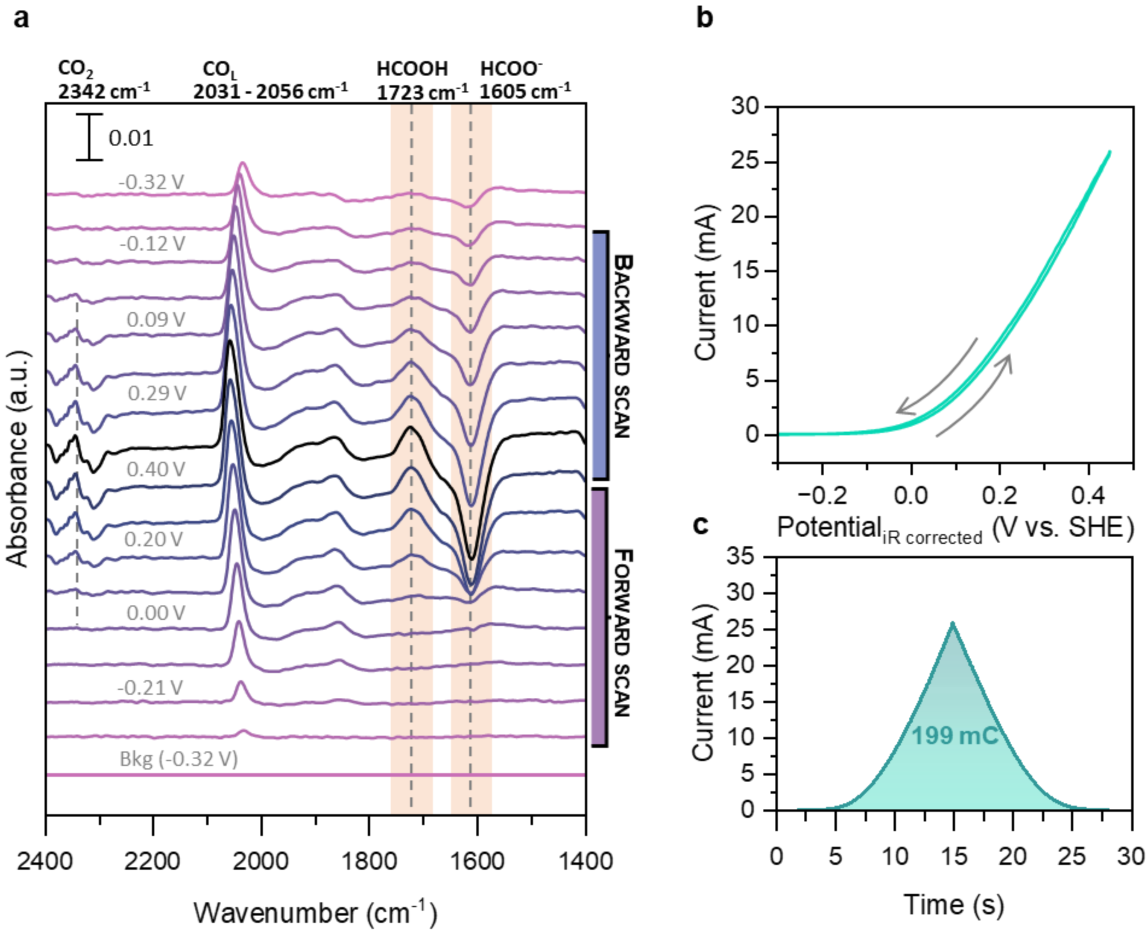

Figure S21. Forward and backward scan of *in situ* SEIRAS spectra during cyclic voltammetry from -0.32 V to 0.45 V vs. SHE (50 mV/s) in Na[$(HCOO)_{0.25}(ClO_4)_{0.75}$] · 4 H_2O (**a**); a spectrum was acquired every 2.1s. The background spectrum is the first spectrum of the cyclic voltammetry measurement covering the voltage window -0.32 – 0.20 V. (**b**) current response during SEIRAS measurement in Na[$(HCOO)_{0.25}(ClO_4)_{0.75}$] · 4 H_2O. Passed total charge during the forward scan in Na[$(HCOO)_{0.25}(ClO_4)_{0.75}$] · 4 H_2O (**c**).

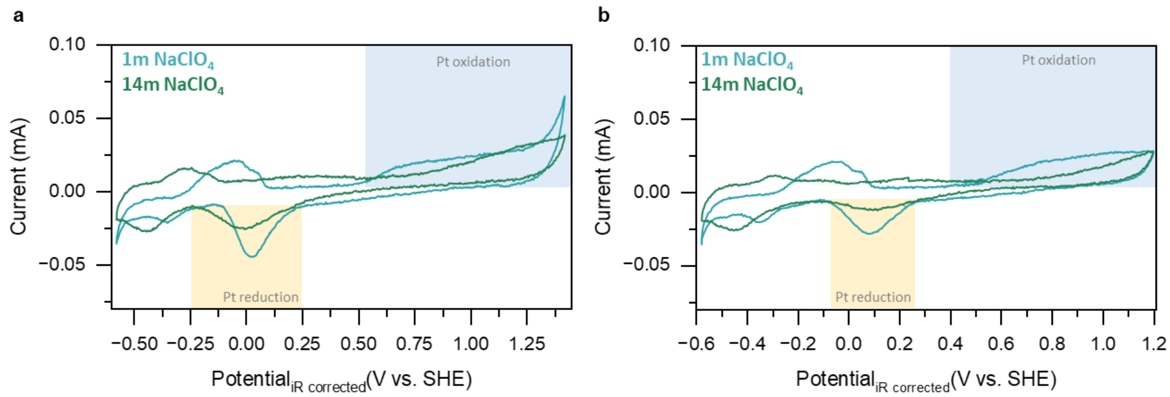

Figure S22. Cyclic voltammetry (100 mV/s) in diluted and WIS perchlorate electrolytes.The upper potential boundary is varied between 1.4 V vs. SHE (**a**) and 1.2 V vs. SHE (**b**) to evaluate the oxidation current of Platinum within the respective boundaries and electrolyte.

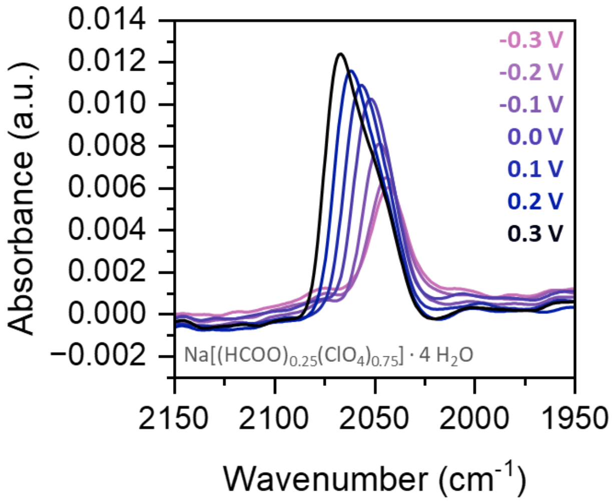

Figure S23. *In situ* SEIRAS spectra of the CO_L region in $Na[(HCOO)_{0.07}(ClO_4)_{0.93}] \cdot 4\,H_2O$ during linear sweep voltammetry (50mV/s) from -0.32 to 0.32 V. A spectrum was taken every 100 mV.

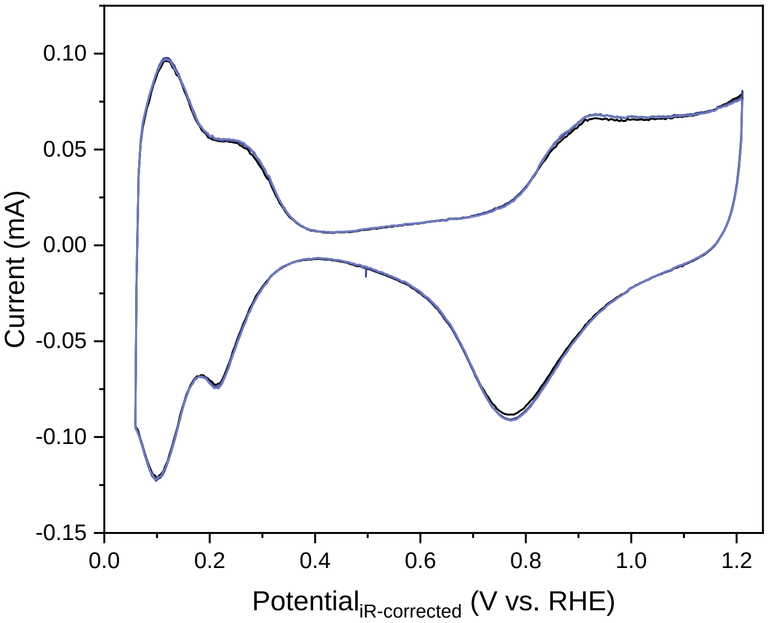

Figure S24. Cyclic voltammetry (500 mV/s) of the used polycrystalline Pt RDE electrode in 0.1 M $HClO_4$. The H-UPD region during cyclic voltammetry was used for the determination of the electrochemical active surface area (ECSA) as previously described.[3] The roughness factor of the RDE electrode was determined to be ~ 1.14.

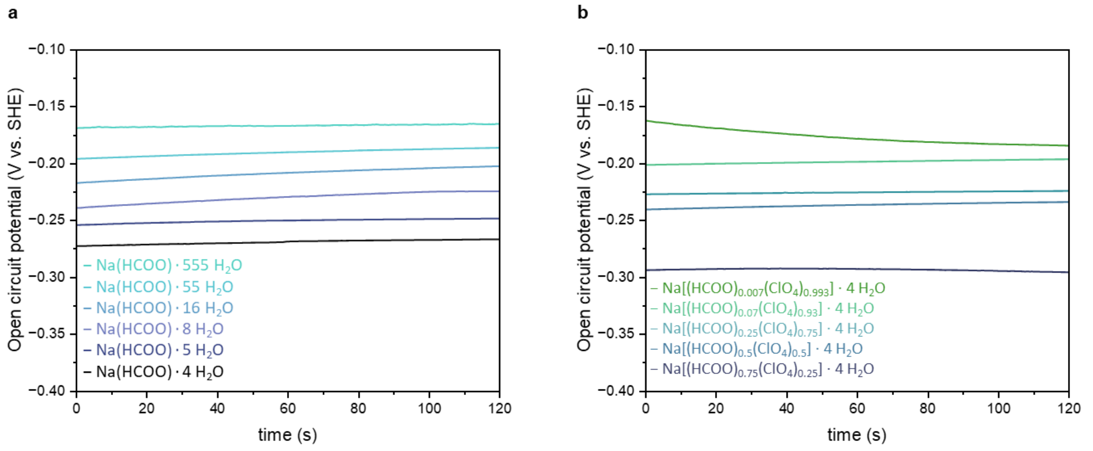

Figure S25. Monitored open circuit potential in each electrolyte before cyclic voltammetry measurements.

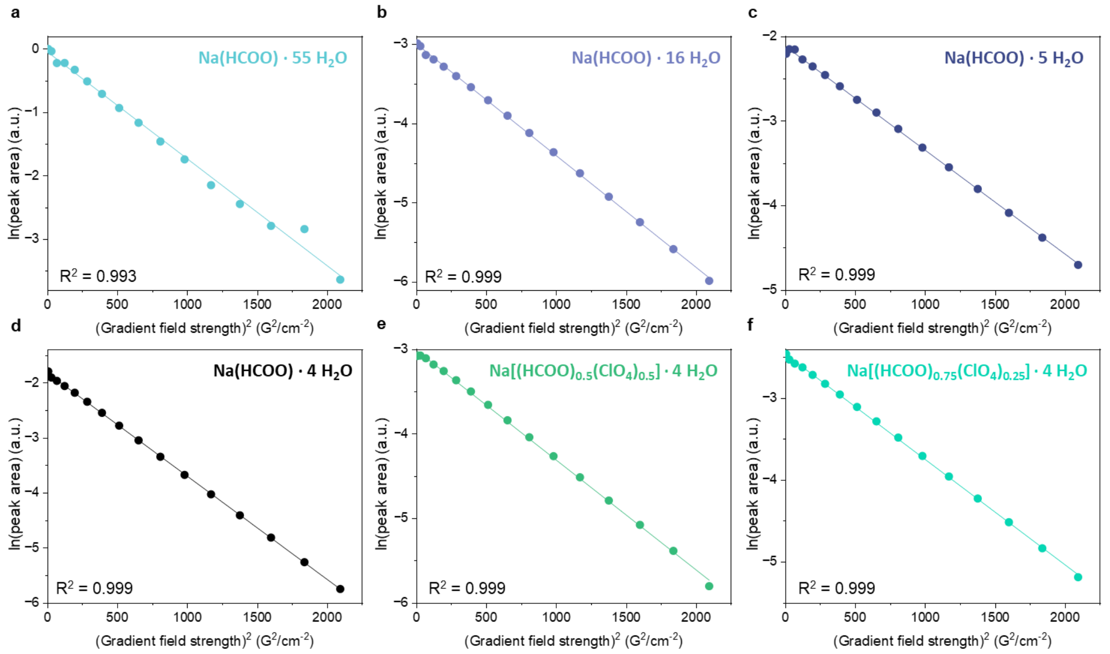

Figure S26. Linear DOSY NMR plot showing ln(peak intensity) as a function of the squared pulsed field gradient strength and the corresponding fit quality based on the Stejskal-Tanner equation (**a-f**).

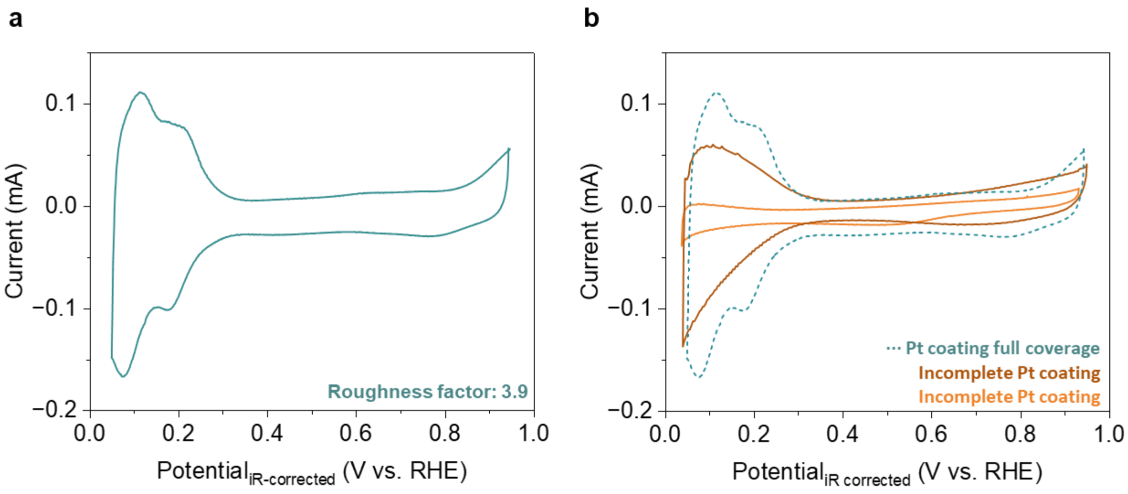

Figure S27. Cyclic voltammetry (50 mV/s) of the thin film Pt electrode used for the SEIRAS experiments in 0.05 M $HClO_4$ (**a**). The roughness factor of the Pt thin film was determined based on the H-UPD region in the CV, as previously reported.[3] Comparison of cyclic voltammetry response (50 mV/s) from incompletely deposited Pt thin films (**b**) with the cyclic voltammetry of the continuous Pt thin film depicted in (a).

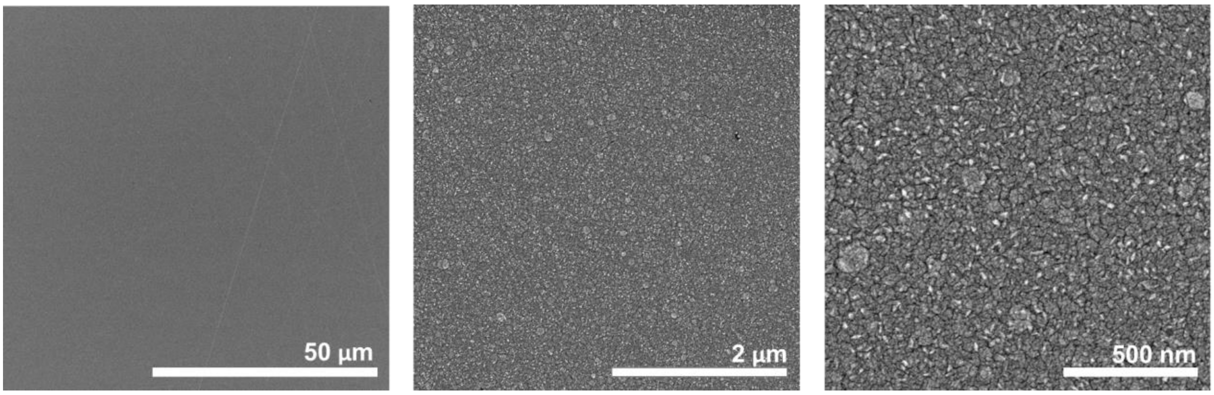

Figure S28. Plan-view SEM pictures acquired at different magnifications from the as-prepared Pt thin film utilized for SEIRAS measurements.

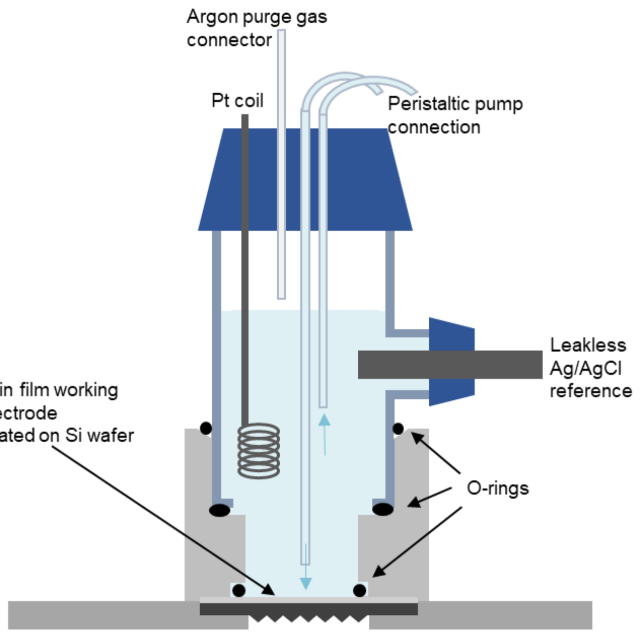

Figure S29. *In situ* SEIRAS cell design. The electrolyte was pumped through the cell to ensure proper convection during the measurement. The pumping speed was limited to 15 ml/min to avoid film delamination of the working electrode.

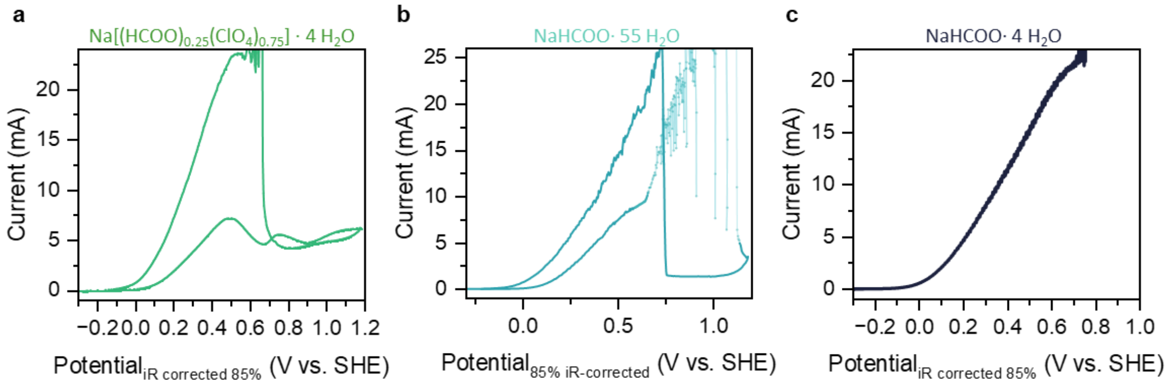

Figure S30. Current response in SEIRAS cell during formate oxidation in $Na[(HCOO)_{0.07}(ClO_4)_{0.93}] \cdot 4\ H_2O$ (a), $NaHCOO \cdot 55\ H_2O$ (b) and $NaHCOO \cdot 4\ H_2O$ (c). The oscillations observed in the current response are typical for formate oxidation when far from equilibrium.[4] FOR peak maxima are shifted to higher potentials in the SEIRAS cell due to enhanced acidification arising from reduced convection, high currents and surface roughness compared to RDE measurements as well as a higher resistance in the SEIRAS cell (~10 Ω vs. ~1 Ω).

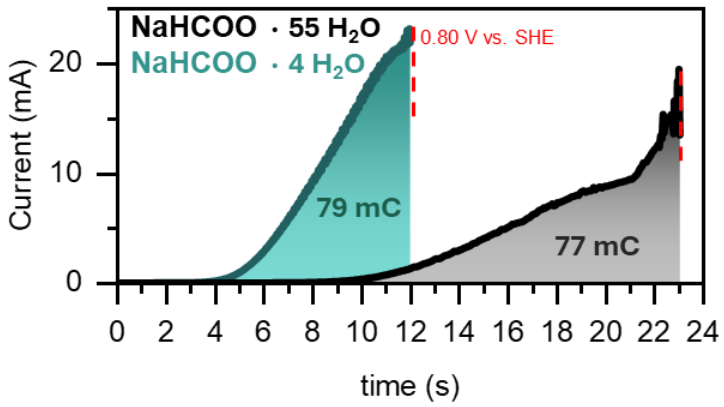

Figure S31. Measured current as a function of time during SEIRAS experiments of the respective electrolytes.

Supplementary tables

Table S1. MD-derived coordination numbers of sodium ions with formate, water and perchlorate. The coordination numbers were taken from the local minima of the rdf function.

Electrolyte	$CN_{(Na^+-HCOO^-)}$ 1st shell (2nd shell)	$CN_{(Na^+-H_2O)}$ 1st shell(2nd shell)	$CN_{(Na^+-ClO_4^-)}$ 1st shell	$CN_{(H_2O-H_2O)}$ 1st shell
$Na(HCOO)\cdot 55\ H_2O$	0.2 (0.8)	5.5 (22.7)	/	3.5
$Na(HCOO)\cdot 16\ H_2O$	0.6 (2.2)	5.1 (20.9)	/	3.0
$Na(HCOO)\cdot 8\ H_2O$	1.4 (4.0)	4.3 (18.0)	/	2.6
$Na(HCOO)\cdot 5\ H_2O$	2.4 (5.6)	3.3 (14.9)	/	2.2
$Na(HCOO)\cdot 4\ H_2O$	2.7 (6.2)	3.0 (13.7)	/	2.1
$Na[(HCOO)_{0.25}(ClO_4)_{0.75}]\cdot 4\ H_2O$	0.7 (1.4)	3.3 (13.3)	1.9	1.8
$Na[(HCOO)_{0.5}(ClO_4)_{0.5}]\cdot 4\ H_2O$	1.4 (2.9)	3.2 (13.4)	1.2	1.9
$Na[(HCOO)_{0.75}(ClO_4)_{0.25}]\cdot 4\ H_2O$	2.1 (4.6)	3.1 (13.4)	0.6	2.0

Table S2. NMR-derived self-diffusion coefficient of formate (D_{HCOO^-}), peak current density (J_{max}) and Viscosity in selected electrolytes.

Electrolyte	Molality	D_{HCOO^-} (cm^2/s)	J_{max} (mA/cm^2)	Viscosity (mPas)
$Na(HCOO)\cdot 55\ H_2O$	1 m NaHCOO	$1.1 \cdot 10^{-5}$	10	1.1
$Na(HCOO)\cdot 8\ H_2O$	7 m NaHCOO	$5.1 \cdot 10^{-6}$	87	3.6
$Na(HCOO)\cdot 5\ H_2O$	10.5 m NaHCOO	$2.3 \cdot 10^{-6}$	95	6.4
$Na(HCOO)\cdot 4\ H_2O$	14 m NaHCOO	$1.6 \cdot 10^{-6}$	96	8.3
$Na[(HCOO)_{0.5}(ClO_4)_{0.5}]\cdot 4\ H_2O$	7 m NaHCOO + 7 m $NaClO_4$	$2.7 \cdot 10^{-6}$	84	10.2
$Na[(HCOO)_{0.75}(ClO_4)_{0.25}]\cdot 4\ H_2O$	10.5 m NaHCOO + 3.5 m $NaClO_4$	$2.2 \cdot 10^{-6}$	119	/

Table S3. Calculated formate/formic acid ratio at different pH values based on the formular denoted in supplementary note 2. A pKa value of 3.75 was used for formic acid.

pH	$\frac{[HCOO^-]}{[HCOOH]}$	$[HCOOH]$ (%)
6.0	178	0.6
5.5	56	1.7
5.0	18	5.3
4.5	6	15.1
4.0	2	36.0
3.75	1	50.0

Table S4. Open circuit potential of the respective electrolytes. The last 10s of the OCV measurement vs. time were averaged for OCV determination, which show a standard deviation below 0.001 V.

Electrolyte	Open circuit potential (V vs. SHE)
$Na(HCOO)\cdot 555\ H_2O$	-0.165
$Na(HCOO)\cdot 55\ H_2O$	-0.187
$Na(HCOO)\cdot 16\ H_2O$	-0.203
$Na(HCOO)\cdot 8\ H_2O$	-0.225
$Na(HCOO)\cdot 5\ H_2O$	-0.249
$Na(HCOO)\cdot 4\ H_2O$	-0.267
$Na[(HCOO)_{0.007}(ClO_4)_{0.993}]\cdot 4\ H_2O$	-0.184
$Na[(HCOO)_{0.07}(ClO_4)_{0.93}]\cdot 4\ H_2O$	-0.196
$Na[(HCOO)_{0.25}(ClO_4)_{0.75}]\cdot 4\ H_2O$	-0.224
$Na[(HCOO)_{0.5}(ClO_4)_{0.5}]\cdot 4\ H_2O$	-0.234
$Na[(HCOO)_{0.75}(ClO_4)_{0.25}]\cdot 4\ H_2O$	-0.295

Table S5. Measured pH values of the Ar-bubbled electrolytes. Each electrolyte was measured four times to determine average and standard deviation.

Electrolyte composition molal (molar)	Electrolyte Molecular denotation	pH value $\pm$ StdeV
0.1 m NaHCOO (0.1 M)	$Na(HCOO)\cdot 555\ H_2O$	7.35 ± 0.06
1 m NaHCOO (0.97 M)	$Na(HCOO)\cdot 55\ H_2O$	7.54 ± 0.08
3.5 m NaHCOO (3.19 M)	$Na(HCOO)\cdot 16\ H_2O$	7.81 ± 0.03
7 m NaHCOO (5.82 M)	$Na(HCOO)\cdot 8\ H_2O$	8.06 ± 0.02
10.5 m NaHCOO (7.95 M)	$Na(HCOO)\cdot 5\ H_2O$	8.20 ± 0.05
14 m NaHCOO (9.63 M)	$Na(HCOO)\cdot 4\ H_2O$	8.28 ± 0.05
0.1 m NaHCOO + 14 m $NaClO_4$	$Na[(HCOO)_{0.007}(ClO_4)_{0.993}]\cdot 4\ H_2O$	8.69 ± 0.02
1 m NaHCOO + 13 m $NaClO_4$	$Na[(HCOO)_{0.07}(ClO_4)_{0.93}]\cdot 4\ H_2O$	7.92 ± 0.04
3.5 m NaHCOO + 10.5 m $NaClO_4$	$Na[(HCOO)_{0.25}(ClO_4)_{0.75}]\cdot 4\ H_2O$	7.86 ± 0.04
7 m NaHCOO + 7 m $NaClO_4$	$Na[(HCOO)_{0.5}(ClO_4)_{0.5}]\cdot 4\ H_2O$	7.81 ± 0.10
10.5 m NaHCOO + 3.5 m $NaClO_4$	$Na[(HCOO)_{0.75}(ClO_4)_{0.25}]\cdot 4\ H_2O$	8.11 ± 0.04
10.5 m NaHCOO + 3.5m NaOTf	$Na[(HCOO)_{0.75}(OTf)_{0.25}]\cdot 4\ H_2O$	9.17 ± 0.04

Table S6. Selected gradient pulse duration and diffusion time for diffusion NMR experiments.

Electrolyte	Gradient pulse duration δ (ms)	Diffusion delay Δ (ms)
Na(HCOO)· 55 H_2O	1.2	158
Na(HCOO)· 8 H_2O	1.8	118
Na(HCOO)· 5 H_2O	1.2	498
Na(HCOO)· 4 H_2O	2.6	247
Na[$(HCOO)_{0.5}(ClO_4)_{0.5}$] 4 H_2O	1.8	198
Na[$(HCOO)_{0.75}(ClO_4)_{0.25}$]·4 H_2O	1.8	248

Table S7. Composition, equilibrated box length *L*, and density ρ_{MD} of the simulated systems.

	H_2O	Na^+	$HCOO^-$	ClO_4^-	*L* (Å)	ρ_{MD} (g/cm³)
Na(HCOO)· 55 H_2O	6600	120	120	-	58.6794	1.04
Na(HCOO)· 16 H_2O	6192	387	387	-	58.4701	1.15
Na(HCOO)· 8 H_2O	5640	705	705	-	58.3789	1.25
Na(HCOO)· 5 H_2O	5100	1020	1020	-	58.5291	1.34
Na(HCOO)· 4 H_2O	4800	1200	1200	-	58.6685	1.38
Na[$(HCOO)_{0.25}(ClO_4)_{0.75}$]·4 H_2O	4800	1200	300	900	60.8206	1.60
Na[$(HCOO)_{0.5}(ClO_4)_{0.5}$]·4 H_2O	4800	1200	600	600	60.1607	1.53
Na[$(HCOO)_{0.75}(ClO_4)_{0.25}$]·4 H_2O	4800	1200	900	300	59.4581	1.46

Supplementary notes

Supplementary note 1. Counter reaction and charge balancing of FOR in WIS electrolytes

The hydrogen evolution reaction is generally considered to proceed as counter reaction during FOR in aqueous solutions with Pt as counter electrode (Pt CE, **Eq. S2**). [1,2] This is further corroborated by the Pt CE potential window (**Figure S8a**) and the absence of other reduction products in NMR (**Figure S8b**).[1,2] The formed hydroxyl ions charge-balance sodium cations (**Eq. S3**) through the formation of sodium bicarbonate. Charge balance was further confirmed by measuring the pH increase after 50 FOR cycles in NaHCOO · 5 H_2O and subsequent dilution of reaction solution to 1 m NaHCOO for accurate pH determination. The pH increased from 7.5 to 8.4 showing net-hydroxide production and alkalinization of the electrolyte, charge-balancing consumed formate.

**Anode:**	$NaHCOO_{aq} \rightarrow CO_2 + H^+ + Na^+ + 2e^-$	(Eq. S1)
**Cathode:**	$2\,H_2O + 2e^- \rightarrow 2OH^- + \mathrm{H}_2$	(Eq. S2)
**Total:**	$NaHCOO_{aq} + H_2O \rightarrow NaHCO_3 + \mathrm{H}_2$	(Eq. S3)

Supplementary note 2: Calculation of formic acid/formate ratio with respect to the pH.

$$pH = pK_a + \log\left(\frac{[HCOO^-]}{[HCOOH]}\right)$$

$$\frac{[HCOO^-]}{[HCOOH]} = 10^{pH - pK_a}$$

Supplementary references

[1] D. Gomez Vazquez, J. Ingenmey, K. Trapp, D. Ciliak, M. Salanne, M. R. Lukatskaya, “Extended Stability Window in Water-in-Salt Electrolytes: Understanding the Origins” *J. Am. Chem. Soc.* **2025**, *147*, 35953–35961.

[2] N. Dubouis, P. Lemaire, B. Mirvaux, E. Salager, M. Deschamps, A. Grimaud, “The role of the hydrogen evolution reaction in the solid-electrolyte interphase formation mechanism for ": Water-in-Salt " electrolytes” *Energy Environ. Sci.* **2018**, *11*, 3491–3499.

[3] Q.-S. Chen, J. Solla-Gullón, S.-G. Sun, J. M. Feliu, “The potential of zero total charge of Pt nanoparticles and polycrystalline electrodes with different surface structure: The role of anion adsorption in fundamental electrocatalysis” *Electrochim. Acta* **2010**, *55*, 7982–7994.

[4] M. J. Salamon, V. Briega-Martos, A. Cuesta, E. Herrero, “Insight into the role of adsorbed formate in the oxidation of formic acid from pH-dependent experiments with Pt single-crystal electrodes” *Journal of Electroanalytical Chemistry* **2022**, *925*, 116886.
